# Extracellular vesicles, from the pathogenesis to the therapy of neurodegenerative diseases

**DOI:** 10.1186/s40035-022-00330-0

**Published:** 2022-12-12

**Authors:** Xiaohuan Xia, Yi Wang, Jialin C. Zheng

**Affiliations:** 1 Center for Translational Neurodegeneration and Regenerative Therapy, Tongji Hospital, Tongji University School of Medicine, Shanghai, 200072 China; 2 Shanghai Frontiers Science Center of Nanocatalytic Medicine, 200331 Shanghai, China; 3 Key Laboratory of Spine and Spinal cord Injury Repair and Regeneration (Tongji University), Ministry of Education, 200065 Shanghai, China; 4 Translational Research Institute of Brain and Brain-Like Intelligence, Shanghai Fourth People’s Hospital, Tongji University School of Medicine, 200434 Shanghai, China; 5 Translational Research Center, Shanghai Yangzhi Rehabilitation Hospital Affiliated to Tongji University School of Medicine, Shanghai, 201613 China; 6 Collaborative Innovation Center for Brain Science, Tongji University, 200092 Shanghai, China; 7 Center for Translational Neurodegeneration and Regenerative Therapy, Tongji Hospital Affiliated to Tongji University School of Medicine, Shanghai, 200065 China

**Keywords:** Extracellular vesicle, Exosome, Neurodegenerative disease, Therapeutics, Biomarker

## Abstract

Extracellular vesicles (EVs) are small bilipid layer-enclosed vesicles that can be secreted by all tested types of brain cells. Being a key intercellular communicator, EVs have emerged as a key contributor to the pathogenesis of various neurodegenerative diseases (NDs) including Alzheimer’s disease, Parkinson’s disease, amyotrophic lateral sclerosis, and Huntington’s disease through delivery of bioactive cargos within the central nervous system (CNS). Importantly, CNS cell-derived EVs can be purified via immunoprecipitation, and EV cargos with altered levels have been identified as potential biomarkers for the diagnosis and prognosis of NDs. Given the essential impact of EVs on the pathogenesis of NDs, pathological EVs have been considered as therapeutic targets and EVs with therapeutic effects have been utilized as potential therapeutic agents or drug delivery platforms for the treatment of NDs. In this review, we focus on recent research progress on the pathological roles of EVs released from CNS cells in the pathogenesis of NDs, summarize findings that identify CNS-derived EV cargos as potential biomarkers to diagnose NDs, and comprehensively discuss promising potential of EVs as therapeutic targets, agents, and drug delivery systems in treating NDs, together with current concerns and challenges for basic research and clinical applications of EVs regarding NDs.

## Introduction

Neurodegenerative diseases (NDs) are a group of disorders characterized by progressive neuronal loss associated with deposition of pathological proteins/peptides in the central and peripheral nervous systems [1]. Examples of NDs include Alzheimer’s disease (AD), Parkinson’s disease (PD), Huntington’s disease (HD), amyotrophic lateral sclerosis (ALS), and many other NDs. The pathogeneses of NDs are complicated and far from being fully understood. However, the delivery of pathogenic molecules among diverse cellular populations and the establishment of disease-associated microenvironment have emerged as key contributors [2, 3]. Although cells can interact with surrounding and distant cells through various pathways, extracellular vesicles (EVs) are one of the most powerful tools for intercellular communication [2, 4, 5].

The discovery of EVs is one of the most groundbreaking discoveries in cell biology over the past few decades [2, 4, 6, 7]. EVs are the nanoscale bilipid layer-enclosed vesicles that are released from most eukaryotic cells and can be found in tissues and biological fluids [8–11]. They are a heterogeneous group of cell-derived membranous structures that mainly comprise exosomes and ectosomes/microvesicles (MVs) [2, 4, 8–12]. Other types of EVs include mitovesicles [12], apoptotic bodies, and retrovirus-like vesicles [13], which are not included in this review. In the central nervous system (CNS), EVs are widely involved in brain development and regeneration by regulating cell fate commitment and neural plasticity [14, 15], in the pathogenesis of NDs *via* transferring disease-associated molecules (e.g., amyloid precursor protein [APP], Tau, cytokines) [10, 16], and in the neuroregeneration/repair in NDs and acute brain damage [17]. Therefore, EVs have been considered as important pathological factors, potential therapeutic targets, and promising therapeutic agents of NDs. In this review, we summarize the pathological roles of EVs in NDs, and comprehensively discuss current progress of basic research and clinical investigations that utilize EVs as potential therapeutics and drug delivery systems for treating NDs.

## EVs: biogenesis, composition, and secretion

### Biogenesis of EVs

**Fig. 1:**
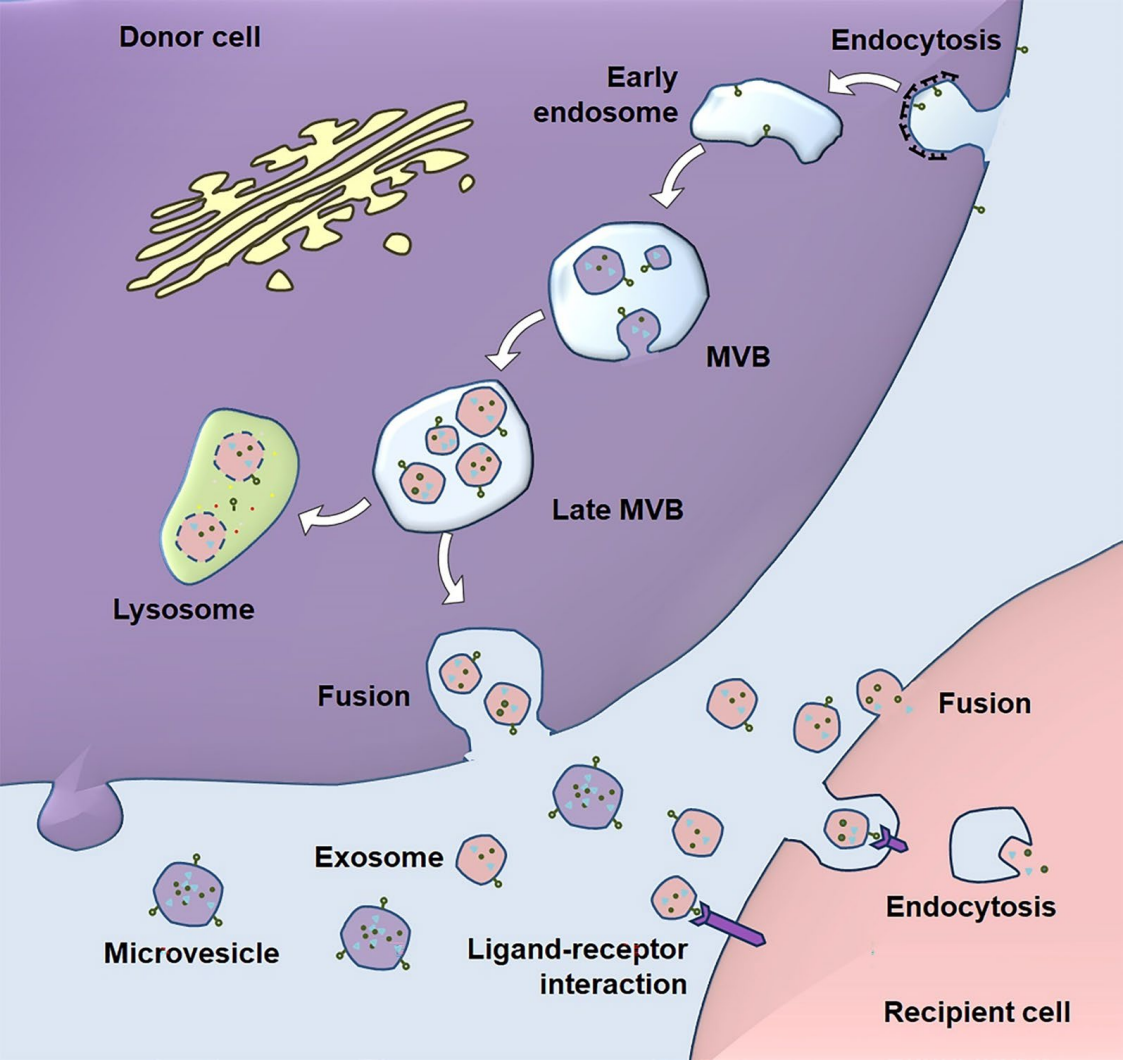
The biogenesis and uptake of EVs. Exosome biogenesis pathway starts with the formation of early endosome by endocytosis at the plasma membrane. ILVs are generated by inward budding of the multivesicular body (MVB, also known as late endosome) lipid bilayer membrane. MVB can fuse with the plasma membrane under the regulation of multistep processes including MVB trafficking along microtubules and docking at the plasma membrane for exosome secretion. Alternatively, MVBs fuse with lysosomes for degradation. Unlike exosomes, microvesicles are released directly by budding from the plasma membrane. EVs in extracellular space bind to the surface of recipient cells through protein-protein or receptor-ligand interactions, leading to the internalization of EVs by recipient cells through fusion or endocytosis. EV contents are then released into the recipient cells to manipulate various biological processes

The mechanism of biogenesis is a major difference between exosomes and MVs. Exosomes are one of the smallest EVs that are also known as endosome-derived vesicles. The biogenesis of exosomes starts with the inward budding of the plasma membrane (Fig. 1). The fusion of primary endocytic vesicles forms early endosome (EE) in clathrin- or caveolin-dependent or -independent pathways [18]. EEs mature into late endosomes, also known as multivesicular bodies (MVBs). The formation and maturation of MVBs is under the regulation of various pathways including Rab5/Rab7 [19] and integral membrane protein of the lysosome/late endosome (SIMPLE) [20]. For instance, Rab5 forms a complex with Rabaptin-5 and Rabex-5, causing rapid recruitment of Rab5 effectors including the VPS34/p150 complex [19]. Rab5 is then removed from MVB membrane by the Mon1/SAND-1/Ccz1 complex, which promotes the recruitment and activation of Rab7 and of the HOPS complex (e.g., Vps11, Vps16, and Vps18) for membrane tethering and fusion [21]. In this process, intraluminal vesicles (ILVs) are formed *via* inward membrane budding in Rab5- and endosomal sorting complex required for transport (ESCRT)-dependent and -independent manners [19, 22–25]. In the ESCRT-dependent machinery, ESCRT-0, ESCRT-I, ESCRT-II, and ESCRT-III constitute the crossroad for recognition of proteins and membrane budding [22–25]. The ESCRT-independent mechanism has been reported in the formation of melanosomes, where Pmel17 [26] and Tetraspanin CD63 [27] contribute to ILV formation. After that, one part of MVBs (degradative MVBs) are degraded by lysosomes, and the remaining MVBs (secretory MVBs) are guided to the plasma membrane [28, 29]. Triggered by Ca^2+^ influx, MVBs fuse with the plasma membrane mainly through the exocytic pathway that requires a fusion machinery (SNARE [soluble N-ethylmaleimide-sensitive factor attachment protein receptor] and tethering factors), molecular switches (small Rab GTPases), cytoskeleton and its motor proteins (actin, microtubules, kinesins, myosins), and other supporting factors [4, 30]. Moreover, recent evidence suggests that only certain subpopulations of MVBs fuse with plasma membrane due to the selective binding of supporting factors for plasma fusion [4, 31, 32]. After MVB fusion with the plasma membrane, ILVs are released into extracellular space, which are defined as exosomes.

Unlike exosomes, MVs are generated through direct outward budding and fission of the plasma membrane. MVs tend to be larger in size (50–2000 nm) compared with exosomes, despite an overlap of the size range. The release of MVs relies on the dynamic interplay between phospholipid redistribution and cytoskeletal protein contraction [13]. In this process, ADP-ribosylation factor 6 activates phospholipase D, leading to the recruitment of extracellular signal-regulated kinase (ERK) to the plasma membrane. ERK phosphorylates and activates myosin light-chain kinase, which triggers the release of MVs.

Notably, although exosomes and MVs utilize distinct pathways for biogenesis, there are many technical challenges for isolating EVs and specific types of EVs due to the small size of EVs and enrichment of contaminants with similar size and/or density as EVs in biological fluids [33, 34]. Several techniques have been developed for isolating EVs (e.g., ultracentrifugation, density-gradient centrifugation, filtration, size-exclusion chromatography, modified polymer co-precipitation, and commercially available commercial kits) and separating exosomes (e.g., immunoaffinity chromatography) [35]. These techniques have their disadvantages such as low purity, high cost, insufficient homogeneity, and high labor intensity [34, 36, 37]. Besides, the characterization of EVs also faces many technical issues. Methodologies that characterize the sizes of EVs like the dynamic light scattering and nanoparticle tracking analyses cannot distinguish nanoscale contaminants from EVs, and bulk methods like western blotting requires a great number of EVs [34]. Inspiringly, single EV-based high-throughput analysis has been developed recently, which opens a window to fully understand the heterogeneity and complex functions of EVs [38, 39].

### Contents of EVs

EVs contain a broad spectrum of proteins, lipids, and nucleic acids. Based on the database ExoCarta (http://www.exocarta.org), there are more than 9700 proteins, 3400 mRNA, and 2800 miRNAs that have been identified in exosomes. Among all identified proteins, exosomes are abundantly loaded with endosome-associated membrane proteins including annexins, flotillin, and Tetraspanins (e.g., CD63, CD81, and CD9) [40, 41]. Exosomes also carry biologically active soluble proteins (e.g., growth factors [42] and transcription factors [43]) and transmembrane proteins (e.g., APP [10]). Furthermore, exosomes contain multiple types of nucleic acids including DNA, mRNAs, and non-coding RNAs like miRNAs and long non-coding RNAs (lncRNAs) [2, 44–46]. These nucleic acids are highly likely to be located within exosomes in a soluble form [47], although recent studies indicated that miRNAs bind to membrane proteins [48]. Besides, exosomes also carry various lipids including sphingomyelin, phosphatidylserine, cholesterol, and ceramide [41, 49], and these lipids are mainly located on the exosomal surface to regulate the biogenesis and release of exosomes [49, 50].

Similar to exosomes, MVs also contain various proteins, lipids, and nucleic acids. Interestingly, MVs have been found to contain certain proteins different from exosomes. For example, MVs express CD40, selectins, integrins, and highly likely cytoskeletal proteins due to their plasma membrane origin [51]. Recent studies reported that small MVs also express CD63-like exosomes, but the expression levels of CD81 and CD9 are significantly lower in MVs versus exosomes [52]. Thus, these surface proteins have been widely utilized to distinguish between exosomes and MVs. Besides, the membranes of MVs are highly enriched in cholesterol, phosphatidylserine, and diacylglycerol [51].

### Content sorting mechanisms of EVs

Molecules in the cytosol are sequestrated into ILVs through various machineries. Proteins can be passively loaded and actively transferred into exosomes. ESCRT-related molecules syndecans and syntenin bind to CD63 and Alix through the LYPX9(n)L motif, therefore enhancing EV accumulation of syntenin, clathrin, Alix, CD63, heat shock proteins, and ubiquitinated proteins [53–56]. Protein sorting into exosomes is mediated by tetraspanins [57], Nedd4 family-interacting protein 1 (Ndfip1) [58], SIMPLE [20], and lipid (raft)-related mechanism in an ESCRT-independent manner [59]. For example, exosomal surface proteins CD9 and CD63 bind to CD10, premelanosome protein (PMEL), Epstein Barr virus (EBV) protein, and the latent membrane protein 1 (LMP1) to facilitate the loading of the latter into ILVs/exosomes [27, 57, 60]. Similar to the situation of protein loading, exosomes are loaded with nucleic acids, especially miRNAs, through passive packaging and selective sorting mechanisms [61, 62]. Growing studies have identified multiple soluble/membrane-bound RNA-binding proteins that function as “chaperones” to transfer miRNAs into exosomes [48, 62, 63]. hnRNPA2B1 specifically binds to certain miRNAs with GGAG and CCCU motifs and selectively loads these miRNAs into exosomes [62]. Similarly, SYNCRIP enhances the sorting of miRNAs into exosomes by the same motif-binding mechanism as hnRNPA2B1 [63]. Ago2, a key component of the RNA-induced silencing complex (RISC), loads miRNAs into exosomes with high miRNA-binding affinity *via* interacting with endosomal CD63 [48]. Together, exosomes contain various bioactive cargos that are passively packaged or selectively loaded. By delivery of cargos, exosomes exert their biological functions under physiological and pathological conditions.

To date, little is known about how cargos in MVs are loaded. It is highly likely that both passive packaging and selective sorting (e.g., endogenous RNA-modulated miRNA sorting [61] and CD63-mediated protein sorting [60]) act in concert to load cargos into MVs. More studies are needed to advance our knowledge on the content-sorting mechanisms of MVs.

### Uptake of EVs

Generally, EVs deliver their cargos to target cells mainly through two ways, endocytosis and fusion with target cell membrane. Endocytosis is the dynamic internalization of cargos by cells for signaling transduction and nutrient uptake. There are at least five types or pathways of endocytosis, namely, caveolae-dependent endocytosis, clathrin-dependent endocytosis, clathrin and caveolin-independent endocytosis, micropinocytosis, and phagocytosis [64]. Accumulating evidence has demonstrated the relevance of these pathways to EV uptake [65, 66]. Rat pheochromocytoma PC12 cells have been found to utilize both clathrin-dependent endocytosis and macropinocytosis for EV uptake [66]. In another type of tumor cells, colon carcinoma COLO205 cells exploit both caveolae- and clathrin-dependent endocytosis for EV uptake [67]. Moreover, EVs can be internalized into macrophages by phagocytosis, and phagocytosis is a much more efficient way for EV uptake than endocytosis [68]. Importantly, many proteins that are involved in recognition and uptake of viruses, liposomes, and nanoparticles are expressed on EVs [69]. Following studies further demonstrated that endocytosis of EVs is facilitated by receptor-ligand complexes consisting of CD9, CD33, CD62, CD81, CD106, and many other molecules [65]. It is worth noting that although ligand-receptor interaction plays an important role in the endocytosis of EVs, whether this interaction confers targeting specificity to EVs remains inconclusive, with literature providing support for both possibilities.

EVs can also release their cargos in the cytosol of the recipient cells through fusion or hemi-fusion of EV and recipient cell membranes [2, 4]. The fusion of EV hydrophobic lipid bilayers and recipient cell plasma membrane has been found to be mediated by fusogenic SNARE proteins, Rab family, lipid raft-like domains, integrins, and adhesion molecules [70]. However, opposite viewpoints are raised that SNARE proteins should not mediate the fusion of EVs with recipient cells as the cytosolic sides of these two membranes are in opposite orientations. Therefore, the mechanisms that mediate EV-to-recipient cell fusion remain largely unknown, and evidence has implicated EV-to-recipient cell fusion as a minor mechanism for EV uptake in physiological conditions. Interestingly, the plasma membrane of tumor cells exhibit great potential to fuse with EVs in the low-pH tumor microenvironment conditions due to the enhanced rigidity of plasma membrane and increased sphingomyelin [71]. Moreover, once activated, platelets display higher fusion capacity with EV membrane, suggesting the relevance of EV-to-recipient cell fusion to the pathogenesis of diseases [72].

Besides, EVs can modulate intracellular signaling through directly binding to the surface receptors on the recipient cell [70]. For instance, dendritic cell-derived EVs activate T lymphocytes *via* CD40-CD40L interaction [73] and enhance immune responses of bystander dendritic cells through binding to Toll-like receptor ligands on bacterial surface [74]. However, whether these EVs are internalized by the recipient cells through ligand-receptor interaction-mediated endocytosis remains to be clarified.

## Pathological roles of EVs in NDs

**Fig. 2:**
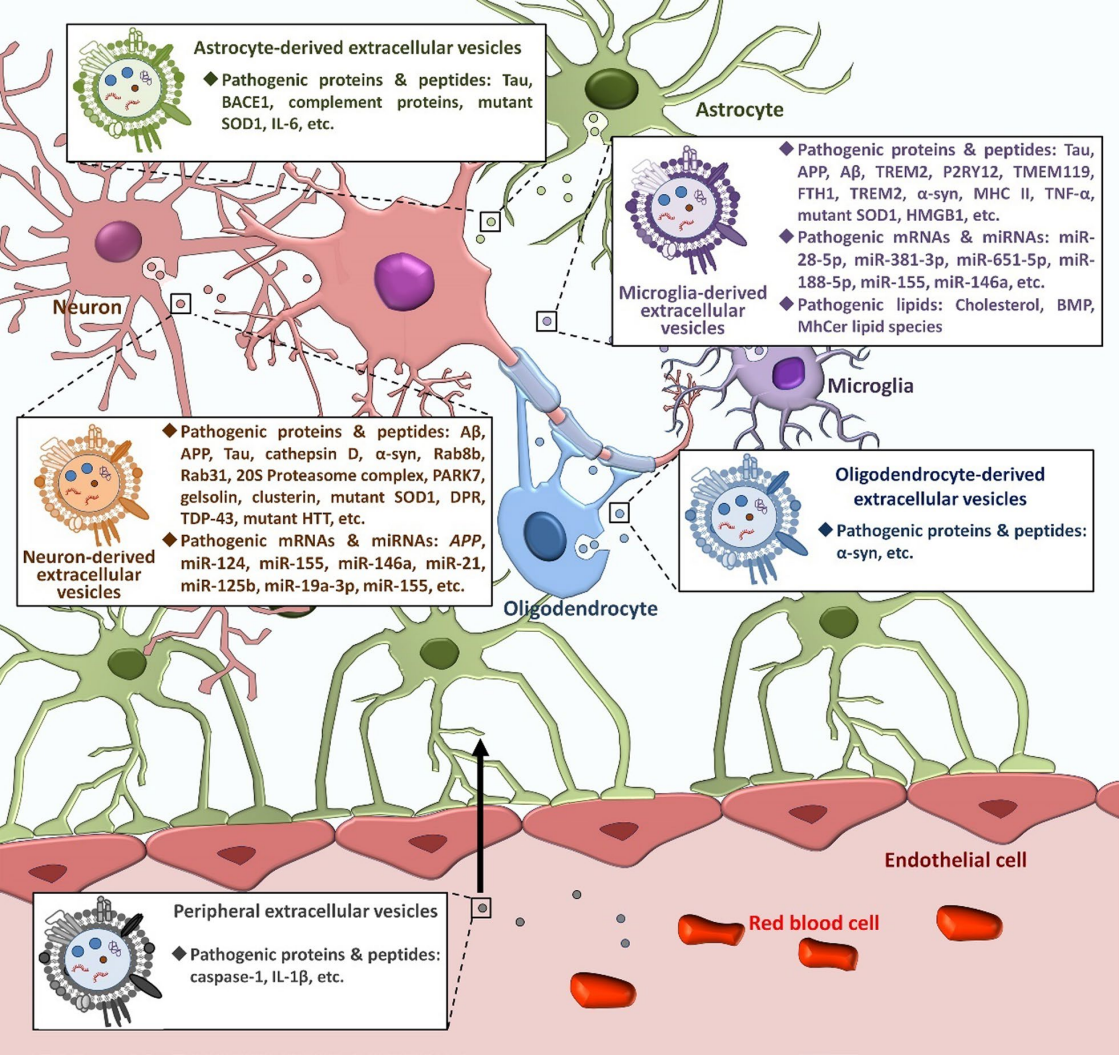
The pathological effects of EVs on NDs. In the brain, there are EVs released from brain cells (e.g., neurons, astrocytes, microglia, and oligodendrocytes) and peripheral EVs that enter the brain through the BBB. Under pathological conditions, these EVs carry pathogenic factors including proteins/peptides, coding and non-coding RNAs, and lipids that contribute to the onset and progression of NDs through facilitating the spreading and aggregation of pathogenic molecules, enhancing cell death, stimulating inflammatory responses, and disrupting the BBB

To date, mounting literature has reported roles of EVs in the occurrence and progression of different NDs including AD, PD, ALS, and HD. Here, we discuss the contributions of EVs derived from different types of brain cells to the pathogenesis of NDs (Table 1; Fig. 2).

**Table 1:** Summary of pathological functions of EVs in NDs

Cell origin	Disease	Up-regulated cargos	Down-regulate cargos	Outcome of EV cargo alteration	References
Neuron	AD	P-T181-tau, Aβ_1−42_, cathepsin D	–	Promoting Aβ deposition and NFT formation	[84]
	AD	APP mRNA & protein	–	Facilitating the production of Aβ	[10]
	AD	miR-124, miR-155, miR-146a, miR-21, miR-125b	–	Inducing microglial activation and pro-inflammatory cytokine release	[87]
	AD	–	miR-185	Elevating APP expression levels	[10]
	PD	α-syn	–	Inducing neurotoxicity and α-syn-rich Lewy bodies formation	[272]
	PD	Rab8b, Rab31	–	Contributing to non-motor symptoms in PD pathology including hearing loss	[123]
	PD	20 S Proteasome complex, PARK7, Gelsolin, Amyloid P component, Clusterin	–	Participating in PD onset and progression	[124]
	PD	miR-19a-3p, miR-155	–	Mediating α-syn-induced inflammatory responses	[121]
	ALS	mutant SOD1	–	Inducing mitochondrial toxicity	[138]
	ALS	DPRs, TDP-43	–	Inducing astrocyte toxicity and neurodegeneration	[139]
	ALS	miR-4736, miR-4700-5p, miR-1207-5p, miR-4739, miR-4505, miR-24-3p, miR-149-3p, etc.	miR-1268a, miR-2861, miR-4508, miR-4507, miR-3176, miR-3911, miR-150-3p, etc.	Disturbing neuroplasticity and enhancing neural damage	[141]
	HD	mutant HTT	–	Inducing neurodegeneration	[156]
Astrocyte	AD	p-Tau	–	Enhancing the formation of NFT	[95]
	AD	BACE1, complement proteins	–	Promoting Aβ cleavage and neuronal damage	[99, 100]
	PD	–	miR-200a-3p	Inhibiting c-Jun N-terminal kinase cell death pathway	[129]
	ALS	mutant SOD1	–	Inducing selective motor neuron death	[146]
	ALS	–	miR-494-3p	Unlocking SEMA3A-induced motor neuron degeneration	[147]
	ALS	IL-6	–	Exacerbating pro-inflammatory responses of neuroglial cells	[148]
	HD	–	CRYAB	Suppressing EV secretion	[273]
Microglia	AD	Human tau	–	Spreading of tau protein	[80]
	AD	TREM2	–	Changing environment around Aβ and promoting microglia to phagocytose Aβ	[114]
	AD	APP/Aβ, P2RY12, TMEM119, FTH1, TREM2	–	Stimulating microglial activation and contributing to Aβ deposition	[111, 112]
	AD	Cholesterol, BMP, MhCer lipid species	DHA-containing polyunsaturated lipids	Inducing defect in acyl-chain remodeling	[112]
	AD	miR-28-5p, miR-381-3p, miR-651-5p, miR-188-5p	–	Enhancing neuroinflammation and cellular senescence	[112]
	PD	α-syn	–	Spreading α-syn oligomers through microglia-neuron α-syn transmission	[130]
	PD	MHC class II molecules, TNF-α	–	Triggering neuroinflammation and dopaminergic neurodegeneration	[131, 132]
	ALS	mutant SOD1	–	Inducing neurotoxicity and motor neuron death	[150]
	ALS	HMGB1, miR-155, miR-146a	–	Activating microglia and impairing mitophagy	[151]
Oligodendrocyte	PD	α-syn	–	Inducing neurotoxicity and formation of α-syn-rich Lewy bodies	[136]

BMP, bis(monoacylglycerol)phosphate; MhCer, monohexosylceramide; DHA, docosahexaenoic acid

### Pathological roles of EVs in AD

AD is the most common neurodegenerative disease and most common cause of dementia in the elderly [75]. The etiology of AD is not clear, which is mainly related to genetic and environmental factors like phosphorylated Tau protein (p-Tau) and amyloid-beta (Aβ) [75]. Other hypotheses/theories including neuroinflammation, gut-brain axis disorder, and metabolic dysfunction have also been proposed [75–78]. Interestingly, growing evidence has shown altered secretion and functions of EVs during the progression of AD [79], and that blocking EV release significantly mitigates AD phenotype [80], revealing the non-ignorable contributions of EVs to the pathogenesis of AD [81].

#### Pathological roles of neuron-derived EVs (NDEVs) in AD

Neurons are electrically excitable cells that communicate with other cells *via* neurotransmission, and are the main component of the CNS [4]. Neurons release a great number of EVs to modulate synaptic activities and regulate cell homeostasis in physiological conditions [4, 82]. The pathological roles of NDEVs in AD are receiving much attention from the scientific community. Recently, an immunoabsorption-based strategy using anti-human L1CAM antibody has been developed to purify NDEVs from blood samples [83]. NDEVs isolated from blood of AD patients demonstrate significantly increased levels of Aβ_1−42_ and p-Tau, and altered lysosomal proteins, when compared with healthy donors [83, 84]. However, recent studies have questioned the utility of L1CAM as a marker of NDEVs [85, 86]. L1CAM is not specifically expressed in neurons, but also in oligodendrocytes in the CNS, immune cells (e.g., T cells, B cells, and monocytes), and endothelial cells. Besides, Norman et al. demonstrated that L1CAM is not associated with EVs in human CSF or plasma. Instead, ND-related proteins (e.g., soluble α-synuclein [α-syn]) in plasma can nonspecifically bind to the anti-L1CAM antibody and are isolated by L1CAM-immunocapture experiment [85]. Hence, advanced methodology is required to purify NDEVs from blood samples without contamination. In addition, altered levels of AD-related miRNAs have been found in EVs derived from human neuroblastoma SH-SY5Y cells stably expressing APP_695_ Swedish mutation (SH_Swe_) and mouse neuroblastoma N2a cells expressing human APP, compared to the responding controls [10, 87]. These observations suggest that NDEVs facilitate the pathological spread of AD-related factors among brain cells and drive Aβ to form amyloid fibrils in the CNS [88, 89].

The altered profiles of cargos also influence the biological functions of NDEVs. Compared with NDEVs isolated from the plasma of healthy individuals, NDEVs isolated from blood of AD patients exhibit significant neurotoxic effects on cultured E18 rat cortical neurons, ascertained by the reduced cell viability determined by MTT assay [90]. The NDEV-induced neuronal damage is likely mediated by the transition of pathogenic molecules of AD such as APP and toxic Aβ oligomers [10, 88]. The complement system, especially the membrane attack complex (MAC), also mediates the pathological effects of NDEVs isolated from blood of AD patients, since CD59, a GPI-anchored cell membrane glycoprotein that inhibits MAC assembly, significantly reduces NDEV-induced neuronal loss [90]. In an in vitro AD model, EVs derived from SH_Swe_ cells can be internalized by microglia, and induce acute and delayed microglial up-regulation of tumor necrosis factor-alpha (TNF-α) and other pro-inflammatory factors that cause neuroinflammation, through delivery of miR-155, miR-146a, miR-124, miR-21 and miR-125b to the microglia [87]. However, we recently found that N2a cells release miR-185-enriched EVs to suppress the expression of APP in recipient N2a cells in vitro, which implies an anti-Aβ deposition role of NDEVs [10]. Therefore, NDEVs exhibit both pathological and beneficial effects, suggesting dynamic changes of NDEVs during the progression of AD.

#### Pathological roles of astrocyte-derived EVs (ADEVs) in AD

Astrocytes are the most abundant glial cells in the CNS and are associated with many functions vital to CNS physiology, including blood-brain barrier (BBB) formation and maintenance, neuroplasticity, neurotransmission, and metabolic regulation [91]. Astrocytes have high capacity for EV release and ADEVs have been shown to be an important contributor to ND pathogenesis [9, 92].

In AD, astrocytes respond to both p-Tau and Aβ, leading to the accumulation of Aβ_42_ protofibrils within astrocytes. The excessive Aβ up-regulates the expression of p-Tau, prostate apoptosis response 4 and ceramide to form giant endosomes for ADEV release in a co-culture system [93]. In contrast, Abdullah et al. reported that Aβ_1−42_ inhibits ADEV release *via* stimulation of the JNK signal pathway in vitro [94]. Although conflicting results have been obtained regarding the effects of Aβ on ADEV secretion, ADEVs have been found to promote Aβ aggregation and interfere with Aβ uptake by neuroglia [95], leading to neuronal loss in AD cells and animal models [96, 97]. Inhibition of Aβ formation in astrocytes by the calcium-sensing receptor signaling antagonist calcilytic NPS 2143 or blockade of exosome secretion by GW4869, a neutral sphingomyelinase2 (nSMase2) inhibitor [98], has been shown to dramatically repress the release of p-Tau-loaded ADEVs or Aβ aggregation, respectively [95]. Moreover, enzyme-linked immunosorbent assay (ELISA) has identified significantly increased levels of BACE1 and complement proteins (e.g., C3b, and C5b-C9 terminal complex) in both plasma- and CSF-isolated ADEVs [99, 100]. BACE1 is a beta-secretase involved in the cleavage of APP to form Aβ peptides, and C3b and C5b-C9 complex may injure neurons directly or indirectly *via* enhancing microglial neurotoxicity [100, 101]. These results suggest that ADEVs regulate Aβ deposition and exert neurotoxicity through transferring Aβ processing enzymes and pro-inflammatory factors in addition to p-Tau. Interestingly, ADEVs may also function as a negative regulator of AD progression since ultrasound-mediated ADEV release alleviates Aβ-induced neurotoxicity in vitro. Whether ADEVs exert beneficial effects on AD in vivo remains to be investigated.

#### Pathological roles of microglia-derived EVs (MDEVs) in AD

Microglia are resident immune-competent cells of the brain, which respond to exogenous and endogenous CNS insults and regulate brain development, neuronal network maintenance, and injury repair. Under pathological conditions, microglia polarize into different phenotypes to exert neurotoxic or neuroprotective functions and the simplest model defines microglial polarization into two main phenotypes: classic M1 activation (pro-inflammatory) and alternative M2 activation (anti-inflammatory) [102]. Transcriptome studies at the single-cell level further indicate that the M1/M2 paradigm is inadequate to summarize microglial phenotypes, since microglia rarely exhibit a significant bias toward either M1 or M2 phenotype in vivo [103]. Instead, distinct microglia subtypes have been identified in physiological and pathological conditions, which reflect the innate dynamic nature of tissue monocytes [104–106]. Thus, microglial polarization is multidimensional with extensive overlap in gene expression rather than a simplified linear spectrum [105]. The activation of microglia, irrespective of the particular polarized state, has emerged as the driving force of neuroinflammation in NDs [107–109]. In this process, microglia release a great number of EVs to mediate delivery of pathogenic molecules, to regulate the functions and viability of brain cells, and to facilitate the establishment of disease-related microenvironment, suggesting an important role of MDEVs in the pathogenesis of NDs [110].

In AD, MDEVs have been found to directly transfer classic AD pathogenic factors including Aβ and tau among cells. Extracellular Aβ_42_ protofibrils can be internalized by microglia and then trafficked into MDEVs [111–113]. Moreover, MDEVs have been reported to strongly increase Aβ neurotoxicity through promoting Aβ_1–42_ extracellular aggregates to form small soluble neurotoxic species *via* lipid components of EVs. Microglia also phagocytose and load human tau into MDEVs [80]. MDEVs thus deliver tau to neurons through non-synaptic pathways and trigger abnormal aggregation of tau, demonstrating a synergy between microglia and EVs in the spread of tau pathology in human brains [80].

Besides Aβ and p-Tau, shotgun proteomics studies have demonstrated a significant decrease in the abundance of homeostatic microglia markers P2RY12 and TMEM119, and increased levels of AD-associated factors FTH1 and TREM2 in CD11b^+^ MDEVs isolated from cryopreserved human brain tissues of AD patients, compared with age-matched normal/low pathology cases [112]. Lipidomic analysis also showed increases in levels of cholesterol, major bis(monoacylglycerol)phosphate, and monohexosylceramide lipid species, and a significant decline in levels of docosahexaenoic acid-containing polyunsaturated lipids in AD patient brain-derived MDEVs versus controls, indicating potentially defective acyl-chain remodeling [112]. Inflammation and cellular senescence-related miRNAs, namely miR-28-5p, miR-381-3p, miR-651-5p, and miR-188-5p, have also been found to be enriched in AD patient brain-derived MDEVs, further suggesting the complicated mechanisms of MDEV-mediated neuroinflammation and neurotoxicity in AD [112].

Importantly, Li et al. utilized IL-4 to induce the M2 phenotype of microglia and found that EVs derived from M2 microglia restored viability and mitochondrial dysfunction of neuronal cells in vitro, and reduced Aβ deposition in vivo, suggesting the beneficial effects of MDEVs on AD. Moreover, Huang et al. reported that TREM2 on the surface of MDEVs binds to Aβ, thus changing the inflammatory environment around Aβ and facilitating Aβ phagocytosis by microglia, which suggests a MDEV-mediated mechanism of microglia–Aβ crosstalk that accelerates Aβ elimination [114].

Overall, EVs have been identified as a key component of pathological microenvironment in AD as deregulation of EV release and cargo sorting significantly influences the onset and progression of AD. Inspiringly, a great number of studies has been performed and more pathological functions of EVs are highly likely to be announced shortly, making EVs and their contents a potential biomarker and therapeutic target of AD.

### Pathological roles of EVs in PD

PD is another common ND among the elderly with the impairment of voluntary motor control evolving over time. The main pathological change of PD is the degeneration of dopaminergic neurons in substantia nigra of midbrain, resulting in significant decrease of dopamine content in the striatum [115]. Although the exact etiology and natural course of PD have yet to be fully clarified, the spreading of neuronal cytoplasmic protein α-syn with the polymorphous and fibrillar conformation by EVs has emerged as a key pathogenic factor that mediates the degeneration of dopaminergic neurons [115].

#### Pathological roles of NDEVs in PD

Multiple groups have reported the existence of α-syn in NDEVs in an in vitro PD model, SH-SY5Y human neuronal cells with α-syn expression [116, 117]. Afterwards, α-syn has been identified in L1CAM^+^ NDEVs isolated from human blood, and NDEVs collected from the plasma samples of PD patients have significantly higher levels of α-syn compared with healthy controls [118, 119]. Although anti-L1CAM-capture of NDEVs may be problematic, these findings implicate the involvement of NDEVs in the transmission of α-syn. This premise is confirmed by Danzer et al., who demonstrated efficient neuron-to-neuron transportation of α-syn oligomers to induce α-syn oligomerization in normal neurons, therefore inducing neuronal death, promoting the spreading of pathological synuclein, and enhancing the disease process [116, 120]. NDEVs also transfer α-syn to microglia and impair microglial autophagy [121]. A following study showed that the sorting of α-syn into EVs is regulated by sumoylation-mediated membrane binding [122].

In addition, FGF2-triggered hippocampal NDEVs are specifically enriched in Rab8b and Rab31 that may contribute to non-motor symptoms in PD pathology including hearing loss [123]. Other PD-related proteins including the 20 S Proteasome complex (PSMA1-3, PSMA5-7, PSMB1, PSMB3, and PSMB5-6), Parkinson’s disease protein 7 (PARK7), Gelsolin, Amyloid P component, Clusterin, and Stromal cell-derived factor 1 (SDF-1) are also identified in PD patient plasma-derived NDEVs [124]. The enrichment of these pathogenic proteins in NDEVs may also participate in the onset and progression of PD directly or indirectly, which requires further investigations. Moreover, multiple miRNAs including miR-19a-3p and miR-155 have been found to be overloaded into NDEVs collected from in vitro PD models and blood samples of PD patients [121, 124]. miR-19a-3p and its family members in NDEVs target various transcripts including those that translate phosphatase and tensin homolog/AKT/mTOR signaling pathway components to suppress autophagy in recipient cells, and miR-155 is a key mediator of α-syn-induced inflammatory responses [121, 124–126]. Therefore, NDEVs facilitate α-syn aggregation and neuroinflammation *via* delivering PD-associated miRNAs to microglia, hence contributing to the onset and progression of PD.

Meanwhile, the beneficial effects of NDEVs on PD have also been found. EVs isolated during dopaminergic neuron differentiation reduce protein levels of interleukin (IL)-6, IL-1β, TNF-α, and reactive oxygen species (ROS) in the substantia nigra of a rodent model of PD, highly likely through wnt5a-mediated neuroinflammation modulation [127]. Hence, similar to the situation in the pathogenesis of AD, NDEVs are also double-edged in the pathogenesis of PD.

#### Pathological roles of ADEVs in PD

In PD, astrocytes and microglia remove extracellular α-syn *via* endocytosis to avoid α-syn accumulation in neurons [48, 49]. Meanwhile, α-syn uptake induces inflammatory response of astrocytes, which causes excessive release of ADEVs. Although there is evidence supporting glia-glia and glia-neuron transfer of α-syn through EVs [128], whether ADEVs contain α-syn and directly mediate the spreading of α-syn remain unknown. Moreover, although astrocytes carrying PD-related mutant *LRRK2* G2019S release comparable numbers of EVs versus normal astrocytes, the *LRRK2* G2019S-ADEVs fail to provide full neurotrophic support after being internalized by dopaminergic neurons, indicating that alterations of the enrichment of ADEV cargos directly contribute to the progression of PD. miRNAs in ADEVs are a convincing example. Shakespear et al. reported that ADEVs contain high levels of miR-200a-3p which targets the 3’-untranslated region (UTR) of *Map2k4* and *MKK4* mRNA, therefore inhibiting the c-Jun N-terminal kinase cell death pathway in an in vitro model of PD [129]. EVs derived from MPP (a PD-related neurotoxin)-stimulated astrocytes contain reduced levels of miR-200a-3p, resulting in absence of caspase-3 signaling inhibition and enhancement of dopaminergic neuron degeneration.

#### Pathological roles of MDEVs in PD

Investigations on the pathological effects of MDEVs on PD initiated from identification of α-syn oligomers in MDEVs. EVs obtained from microglia treated with preformed fibrils (PFF) (PFF-MDEVs) contain high levels of α-syn oligomers [130]. More importantly, α-syn oligomers have been detected in CD11b^+^ MDEVs derived from CSF of PD patients, confirming the in vitro findings [130]. MDEVs then spread α-syn oligomers through microglia–neuron α-syn transmission, leading to dopaminergic neuron degeneration and behavioral changes of mice that received stereotaxic injection of PFF-MDEVs into the striatum [130]. Moreover, α-syn induces an increase of exosomal secretion by microglia, forming a vicious cycle to exacerbate MDEV-mediated pathological spread of α-syn [131]. Besides, EVs derived from microglia stimulated by α-syn/interferon-γ (IFN-γ)/lipopolysaccharide (LPS) to mimic PD inflammatory conditions also contain high levels of MHC class II molecules and TNF-α that trigger dopaminergic neurodegeneration, indicating the complex mechanisms of MDEV-mediated onset and progression of PD [131, 132].

#### Pathological roles of oligodendrocyte-derived EVs (ODEVs) in PD

Besides the aforementioned cell types, other types of brain cells also perform vital physiological and pathological functions in the brain, particularly oligodendrocytes, glial cells that generate myelin sheaths to promote rapid neurotransmission in the CNS. Triggered by neuronal signals, myelinating oligodendrocytes secrete EVs into the extracellular space [133]. These ODEVs can be internalized by neurons, supporting axonal transport and maintenance [134]. Given the great impact of ODEVs on the homeostasis of the CNS, studies on the pathological contributions of ODEVs in PD have been carried out recently.

The most recent study using a modified ELISA assay for brain-derived EVs, has demonstrated that the plasma levels of ODEVs are significantly higher in PD patients, compared with healthy controls and patients with multiple system atrophy (MSA), a synucleinopathy whose symptoms largely overlap with that of PD [135]. Similar to the cell type-specific EV immunoprecipitation approach for NDEV and ADEV isolation from human blood and CSF, Dutta et al. utilized an antibody for myelin oligodendrocyte glycoprotein (MOG) to collect ODEVs from the blood of PD patients [136]. The collected EVs contain significantly higher levels of α-syn than healthy controls, indicating ODEVs as a platform for α-syn spreading in the CNS [136]. However, our knowledge on the pathological contributions of ODEVs in PD remains seriously lacking, and extensive investigations are needed in the future.

Together, numerous studies have suggested exosomes as a double-edged sword in PD. More comprehensive studies are needed to clarify the pathological and beneficial effects of exosomes on PD.

### Pathological roles of EVs in ALS

ALS is a fatal, adult-onset neurodegenerative disease characterized by a progressive loss of motor neurons in the brain, brainstem, and spinal cord, rapidly leading to atrophy of bulbar, limb, or respiratory muscles. Although the majority of clinical ALS cases are sporadic, mutations in human copper-zinc superoxide dismutase (*SOD1*) and other genes have been identified in inherited cases of ALS. As a key component of pathological microenvironment, EVs have been found to play a significant role in the pathogenesis of ALS.

#### Pathological roles of NDEVs in ALS

One important contribution of NDEVs to the pathogenesis of ALS is the delivery of pathogenic factors to neuroglial cells. A recent study reported that EVs positive for SNAP25 (a synaptic marker) harvested from the brain and spinal cord tissues of an ALS mouse model contain misfolded neurotoxic SOD1 [137]. Microglial uptake of mutant SOD1-containing NDEVs induces inflammatory responses and reduces the phagocytic ability of microglia [138]. Moreover, other pathogenic factors of ALS, dipeptide repeat proteins (DPRs) and TAR DNA-binding protein-43 (TDP-43), have also been found in EVs released from spinal motor neurons derived from induced pluripotent stem cells from C9orf72-ALS patients [139]. DPR-containing NDEVs can be internalized by astrocytes and induce astrocyte toxicity, therefore causing neurodegeneration [139, 140]. These observations suggest a tight association of NDEVs with progressive propagation of ALS-related pathology spreading from the CNS foci. The miRNA profiles are also significantly altered in NDEVs in plasma of ALS patients [141]. In ALS patient plasma-isolated NDEVs, 13 miRNAs were significantly up-regulated (e.g., miR-24-3p) and 17 miRNAs were significantly down-regulated (e.g., miR-150-3p), compared with controls. miR-24-3p has been identified as a neurodegeneration-related miRNA by disturbing neuroplasticity and enhancing neural damage presumably through regulating BOK and CHD5 [142, 143]. In contrast, miR-150-3p has neuroprotective effects by targeting CASP2 [144]. The up-regulated neurotoxic miRNAs and down-regulated neuroprotective ones in NDEVs imply another potential mechanism of NDEV-mediated pathogenesis of ALS. Moreover, the expression levels of proteins that are involved in the regulation of synaptic membrane and axoneme are also significantly reduced in EVs collected from the cerebrospinal fluid (CSF) of ALS patients [145]. However, whether these observations are mediated by NDEVs remains unknown since the cellular origins of these exosomes are unclarified.

#### Pathological roles of ADEVs in ALS

Under ALS pathological conditions, astrocytes exhibit distinct EV secretion capacity. For example, in ALS models in vitro, astrocytes with *SOD1* mutation release more EVs compared with controls [146], leading to increased effect of ADEVs on the brain microenvironment in ALS. More importantly, the content profiles of ADEVs are also significantly altered in ALS. Basso et al. reported that mutant SOD1 is packaged into ADEVs [146]. The delivery of mutant SOD1 from astrocytes to neurons *via* ADEVs induces selective motor neuron death in vitro. Moreover, human induced astrocytes from ALS patients carrying *C9orf72* mutations release EVs lacking miR-494-3p, a negative regulator of axonal maintenance-related gene semaphorin 3 A (*SEMA3A*) [147]. The depletion of miR-494-3p in ADEVs therefore unlocks SEMA3A-induced motor neuron degeneration in ALS. Similar to the situation in vitro, Chen et al. showed a significant increase of IL-6 in ADEVs isolated from the plasma of sporadic ALS patients, suggesting alterations of ADEV cargos in ALS patients [148]. This finding implies an important role of the ADEV-mediated pathological spread of pro-inflammatory factors in the initiation and exacerbation of neuroinflammation, a key pathological feature of ALS.

#### Pathological roles of MDEVs in ALS

The involvement of microglia in the onset and progression of ALS is being increasingly recognized. In ALS animal models, the overexpression of mutant SOD1 drives microglial activation, autophagy impairment, and hyperexpression of pro-inflammatory factors (e.g., MFG-E8, RAGE, IL-1β, TNF-α, and iNOS), therefore reducing the capacity for mutant SOD1 elimination [149]. Consequently, microglia release excessive mutant SOD1 *via* MDEVs [150]. When motor neurons internalize MDEVs, the intracellular accumulation of mutant SOD1 then induces neurotoxicity and neuronal damage [149, 150]. Furthermore, the levels of HMGB1, miR-155 and miR-146a are significantly increased in EVs derived from mutant SOD1-overexpressing microglia [151]. The HMGB1/RAGE axis has been reported to mediate neuroinflammation *via* impairing the mitophagy flux in microglia [152], and miR-155 and miR-146a have been identified as pro-inflammatory miRNAs that regulate microglial activation [153, 154]. Thus, MDEVs enriched in these pro-inflammatory molecules also contribute to neuroinflammation, leading to aggravation of ALS phenotypes.

### Pathological roles of EVs in HD

HD is a rare, progressive, and fatal hereditary ND caused by CAG expansion in the first coding exon of the *HTT* gene [155]. It is characterized by progressive movement dysfunction and cognitive decline, ending in death within 15–20 years after diagnosis. Elevated levels of total HTT and mutant HTT (mHTT) fragments have been reported in EVs from plasma of both pig models and HD patients compared to controls, implying the involvement of EVs in the pathogenesis of HD [156].

#### Pathological roles of NDEVs in HD

Neurons express excessive HTT in the brains of HD patients [155, 157]. EVs have been found to inherit the mRNA with an expanded CAG-repeat element from their parent cells with excessive HTT expression, although total HTT and mutant HTT fragments have not been detected in NDEVs [157]. These observations implicate that NDEVs participate in the spreading of pathogenic HTT within the brain, although conclusive evidence remains lacking. Besides, an in vitro study also suggests a role for NDEVs against HTT spreading [158]. NDEVs can transfer HTT-targeting miRNAs to HD patient-derived neurons, which leads to the inhibition *HTT* mRNA expression in the latter, providing evidence for NDEV-dependent HTT suppression mechanisms [158]. Despite these preliminary studies in vitro, more studies are required to further clarify the pathological/beneficial roles of NDEVs in HD.

#### Pathological roles of ADEVs in HD

To date, studies that focus on the involvement of ADEVs in the pathogenesis of HD remain limited. Deep sequencing analysis of genes highly expressed in ADEVs reveals that ADEVs are responsible for promoting HD [159]. In the HD 140Q knock-in mouse model of HD, although mHTT is not identified in ADEVs, it inhibits ADEV release through suppressing the expression of αB-Crystallin (CRYAB), a heat shock protein that mediates EV secretion [160]. Furthermore, the sorting of CRYAB into ADEVs is also inhibited by mHTT, leading to neuroglial activation and neuroinflammation that cause neurodegeneration in HD.

### Pathological/beneficial roles of peripheral EVs in NDs

Interestingly, growing evidence has implicated the involvement of peripheral EVs in the pathogenesis of neurological diseases with the discovery of crosstalk between brain and other organ systems in a “bottom-up” manner including gut-brain, lung-brain, and bone-brain axes [161, 162]. Intestinal epithelial cells have been reported to release EVs to induce IL-1β-mediated neuronal injury in sepsis-associated encephalopathy, which launches long-term cognitive deficits and neurodegeneration [163]. Moreover, ventilation-induced lung injury causes lung inflammation, leading to selective loading of caspase-1 into lung-derived EVs [164]. Caspase-1-enriched peripheral EVs induce microglial activation and cell pyroptosis in the brain, revealing circulating EVs as a pathogenic factor of NDs [164].

Besides, peripheral EVs have been reported with potential beneficial effects on NDs. For instance, young osteocytes, the most abundant cells in bone, secrete neuroprotective EVs to enhance cognitive function and ameliorate pathological changes in AD mice [161]. Another example is mesenchymal stem cells (MSCs) that have been widely used for production of EVs with therapeutic effects on NDs (details in this field summarized in a later section) [165–167]. These observations imply that endogenous MSCs may release EVs to decrease the risk of NDs or delay the progression of NDs, which is an interesting topic for future investigations.

It is worth noting that there are also hints for the involvement of EVs in the pathogenesis of multiple sclerosis (MS), an autoimmune ND [168]. However, they are not discussed in this review due to the limited literature support. Besides, although out of the scope of our review, EVs also participate in acute neural damage by modulating the activation of neurotoxic microglia and astrocytes [165, 169]. Overall, current evidence indicates both pathological and beneficial roles of EVs in the pathogenesis of NDs. More studies, especially in vivo ones, are urgently needed to clarify the involvement of EVs in NDs, and develop novel EV-based diagnostic and therapeutic strategies for NDs.

## EVs as novel biomarkers for the diagnosis of NDs

Identification of biomarkers for NDs in the blood is challenging since the BBB prevents free passage of molecules between the CNS and blood compartments. Furthermore, several potential biomarkers related to the pathology of NDs are expressed in non-CNS tissues, significantly confounding their measurement in the blood. Given the pathological roles of EVs in NDs and their BBB penetration capacity, the brain-derived EVs natively possess the potential to serve as biomarkers for diagnosis of NDs. In this section, we summarize recent studies that provide evidence for utilizing EVs and their cargos as potential biomarkers for disease diagnosis (Table 2).

**Table 2:** Summary of differentially expressed EV contents and their potential diagnostic values in NDs

Disease	Down-regulated	Up-regulated	AUC	Sensitivity (%)	Specificity (%)	Specimens	Species	References
AD	APOC3, APOH, C4BPα, CO3, KV230	AACT, CO9, IGHM, K2C6A	–	–	–	Serum EVs	Human	[172]
AD	miR-342-3p, miR-23b-3p, miR-24-3p, miR-125b-5p	miR-141-3p, miR-342-5p	0.919	81.7	–	Serum EVs	Human	[174]
AD	BACE-1-AS-LncRNA (in Pre-AD)	BACE-1-AS-LncRNA (in late AD)	–	75 (pre-AD) 68 (late-AD)	100 100	Plasma EVs	Human	[177]
AD	miR-135a, miR-384	miR-193b	–	99	95	Plasma EVs	Human	[175]
AD	miR-138-5p, miR-342-3p	miR-29c-5p, miR-143-3p, miR-335-5p, miR-485-5p	0.880	–	–	Serum EVs	Human	[176]
AD	–	Aβ_42/40_ miR-384	0.973 0.909	–	–	Blood NCAM^+^ NDEVs	Human	[178]
AD	–	miR-29c-3p	0.927			Blood NCAM^+^ NDEVs	Human	[179]
AD	miR-212 miR-132	–	0.84 0.77	92.2	69	Blood L1CAM^+^ NDEVs	Human	[180]
AD	–	tau, p-T181-tau, p-S396-tau, Aβ_1−42_	0.99	96		Blood L1CAM^+^ NDEVs	Human	[181]
PD	–	α-synuclein	0.724	76.8	53.5	Plasma EVs	Mouse	[183]
PD	Prp	–	–	–	–	Plasma EVs	Human	[184]
PD	miR-1 miR-19b-3p	miR-153, miR-409-3p miR-10a-5p let-7 g-3p	0.920 0.705 0.990 0.900	–	–	CSF EVs	Human	[185]
PD	–	α-synuclein, clusterin	0.98 (PD VS. atypical PD)	–	–	Serum EVs	Human	[119]
PD	α-synuclein (in PD VS. MSA)		0.902 PD VS. MSA)	–	–	Blood MOG^+^ ODEVs, L1CAM^+^ NDEVs	Human	[136]
ALS	–	CORO1A	–	–	–	Plasma EVs	Human	[186]
ALS	–	TDP-43, NFL	–	–	–	Plasma EVs	Human	[187]
ALS	miR-146a-5p	–	–	–	–	CSF EVs	Human	[189]
ALS	–	miR-15a-5p miR-193a-5p	0.976 0.844	92.9 80.0	91.7 88.9	Plasma EVs	Human	[190]
ALS	miR-10b-5p miR-29b-3p	miR-146-5p miR-199a-3p, miR-199a-5p miR-151a-3p, miR-151a-5p	–	–	–	Plasma L1CAM^+^ NDEVs	Human	[191]

AACT, alpha-1-antichymotrypsin; CO9, complement component 9; IGHM, immunoglobulin heavy constant mu; K2C6A, keratin, type II cytoskeletal 6 A; APOC3, apolipoprotein C-III; APOH, beta-2-glycoprotein 1; C4BPα, C4b-binding protein alpha chain; CO3, Complement C3; KV230 immunoglobulin kappa variable 2–30

### EVs as novel biomarkers for the diagnosis of AD

As accumulation of Aβ deposits and formation of neurofibrillary tangles composed of p-Tau in the brain are major pathological hallmarks of AD, neuroimaging approaches including magnetic resonance imaging (MRI) and positron emission tomography (PET), and CSF examinations that detect Aβ (Aβ_1–42_ and Aβ_1–40_) and p-Tau, are used as the gold-standard for AD diagnosis [170]. However, the invasive nature of procedures, the associated risks, and the relatively high costs have limited their practicability. Blood-based diagnostics can overcome these disadvantages due to their non-invasiveness, lower cost, and capability of multiple sampling in large cohorts. The correlation between blood-based AD biomarkers and pathological changes in the brain has been widely investigated [171].

Scientists have isolated EVs from sera of healthy controls and AD patients, and characterized their contents *via* proteomic analyses [172]. They identified that four circulating EV proteins, including alpha-1-antichymotrypsin (AACT) isoform 1, complement component 9, immunoglobulin heavy constant mu Isoform 2, and keratin, type II cytoskeletal 6 A, are significantly up-regulated in AD patients compared with control individuals. Furthermore, five circulating EV proteins, including apolipoprotein C-III, beta-2-glycoprotein 1, C4b-binding protein alpha chain (C4BPα), complement C3, and immunoglobulin kappa variable 2–30 are significantly down-regulated in AD patients compared with control individuals, implying these proteins as putative biomarker candidates. The altered expression levels of two Aβ-binding proteins AACT and C4BPα, in AD patient serum-isolated EVs, were further validated in individuals from independent cohorts [172]. Besides, non-coding RNAs in peripheral EVs were found to have diagnostic potentials for AD [173]. Lugli et al. identified seven miRNAs (e.g., miR-342-3p, miR-141-3p, miR-342-5p, miR-23b-3p, miR-24-3p, miR-125b-5p, and miR-152-3p) in plasma EVs as significant predictors of AD in a machine learning model [174]. The receiver operating characteristic (ROC) curve analysis, which identifies optimal cut-off values for these miRNAs by the area under the curve (AUC), suggested excellent sensitivity of these miRNAs in plasma EV for discriminating AD patients from healthy controls (sensitivity, 81.7%). In addition, Yang et al. reported that miR-135a and miR-384 were up-regulated, while miR-193b was down-regulated in EVs isolated from AD patient sera. The combination of miR-135a, miR-193b, and miR-384 in serum-derived EVs performs better in AD diagnosis than each individual miRNA (sensitivity, 99%; specificity, 95%) [175]. Moreover, miRNAs in blood-derived EVs have been demonstrated to be predictors of AD at the asymptomatic stage (pre-AD). A multicenter study has identified a panel of miRNAs that are changed (up-regulated: miR-29c-5p, miR-143-3p, miR-335-5p, and miR-485-5p; down-regulated: miR-138-5p and miR-342-3p) in AD patients and predicted that this panel can detect pre-AD 5 to 7 years before the onset of cognitive decline (AUC = 0.88) [176]. Fotuhi et al. also found that the level of BACE1-AS lncRNA in plasma-derived EVs significantly differs between AD patients and healthy controls, and that the plasma-derived EV lncRNA BACE1-AS exhibited great diagnostic power for pre-AD (sensitivity, 75%; specificity, 100%) [177]. These findings show the possibility of utilizing circulating EV contents as a biomarker for AD before the occurrence of clinical symptoms.

To further enhance the sensitivity and specificity of EV-based diagnosis, scientists have made a great effort to identify potential AD biomarkers in NDEVs, ADEV, and MDEVs isolated from plasma or serum. They demonstrated that Aβ_42/40_ (AUC = 0.973) and miR-384 (AUC = 0.909) in NDEVs co-labeled with neural cell adhesion molecule (NCAM) and ATP-binding cassette transporter A1 have potential advantages in AD diagnosis [178]. In another study, miR-29c-3p in plasma NCAM/amphiphysin 1 dual-labeled NDEVs showed a good diagnostic performance for subjective cognitive decline (AUC = 0.789) and AD (AUC = 0.927) [179]. Combination of Aβ_42_, Aβ_42/40_, Tau, p-T181-tau, and miR-29c-3p in plasma-isolated NDEVs displays even better diagnostic efficiency than each individual biomarker. More importantly, the levels of these AD biomarkers in plasma-isolated NDEVs are strongly correlated to those in the CSF, and the AD biomarkers of the two sources have comparable diagnostic power (plasma-isolated NDEVs, AUC = 0.911; CSF-isolated NDEVs, AUC = 0.901). Cha et al. showed that miR-212 and miR-132 were down-regulated in AD patient plasma-derived NDEVs and could be used as potential AD biomarkers (AUC = 0.84, sensitivity = 92.2%, specificity = 69.0% for miR-212; AUC = 0.77 for miR-132) [180]. Importantly, isolation of NDEVs from plasma significantly increases the sensitivity for diagnosing AD at pre-AD stage, compared with raw plasma-isolated EVs. Fiandaca et al. found that the mean levels of total Tau, p-T181-tau, p-S396-tau, and Aβ_1−42_ in NDEVs isolated from plasma or serum of AD patients were significantly higher than that of healthy donors even 1 to 10 years before they were diagnosed with AD [181]. Combination of these biomarkers in blood-isolated NDEVs displays promising potential for pre-AD diagnosis (AUC = 0.999, sensitivity = 96%), indicating the ability of NDEVs to predict AD onset and development. In addition, accumulating evidence suggests that mitochondrial dysfunction is associated with the contribution of diabetes to AD progression and may serve as a potential biomarker to diagnose AD among diabetic patients. Scientists have reported that the levels of NADH ubiquinone oxidoreductase core subunit S3 (NDUFS3) and succinate dehydrogenase complex subunit B (SDHB) are significantly lower in L1CAM^+^ NDEVs isolated from the plasma of type 2 diabetes mellitus (T2DM) patients with AD dementia and progressive mild cognitive impairment (MCI) patients than in cognitively healthy individuals [182]. They also found that the levels of NDUFS3 and SDHB in plasma-isolated NDEV are lower in progressive MCI patients than in stable MCI patients [182]. These results indicate the promise of mitochondrial proteins in plasma-isolated NDEVs as potential diagnostic biomarkers at the earliest symptomatic stage of AD in participants with diabetes, although further studies separating NDEVs from blood samples using more reliable neuronal markers are required to validate these results [182].

Apart from the potential NDEV biomarkers, MDEV may also contain AD biomarkers. Fernandes et al. found that microglia internalize SH_Swe_ cell-released EVs, which are enriched in miR-155, miR-146a, miR-124, miR-21 and miR-125b, and recapitulate the cells of origin [87]. Their data revealed that miR-21 is a consistent biomarker that is found not only in SH_Swe_ cells and SH_Swe_-released EVs, but also in the recipient microglia and MDEVs. This study highlights miR-21 in EVs as a potential biomarker for AD [87].

### EVs as novel biomarkers for the diagnosis of PD

EVs and their contents have also been studied for their potential as biomarkers of PD. The plasma levels of different types of brain-derived EVs are increased in PD compared to control and MSA [135]. AUC values of the ROC curve for plasma-isolated SNAP25^+^ NDEVs, EAAT1^+^ ADEVs, and OMG^+^ ODEVs were 0.82, 0.75, and 0.78, respectively, indicating the capability of the plasma levels of brain-derived EVs as diagnostic biomarkers for PD.

Besides EVs *per se*, the level of α-syn in EVs remains stably increased with PD progression and is positively correlated with the severity of PD, displaying a moderate diagnostic value (AUC = 0.724, sensitivity = 76.8%, specificity = 53.5%) [183]. Moreover, the level of prion protein (PrP), a protein contributing to cognitive decline in PD patients, in plasma-derived EVs, negatively correlates with the cognitive performance of PD patients, suggesting that PrP in circulating EVs might be a potential biomarker for PD patients at risk of cognitive impairment [184]. In addition to EV proteins, Gui et al. identified down-regulated (e.g., miR-1 and miR-19b-3p) and up-regulated miRNAs (e.g., let-7 g-3p, miR-153, miR-409-3p, and miR-10a-5p) in EVs isolated from CSF of PD patients versus controls [185]. Each of the differentially expressed miRNAs in CSF-derived EVs exhibits excellent to moderate diagnostic power for PD (AUC: 0.780–0.920), and a combination of miR-153 and miR-409-3p achieves an AUC of 0.990 [185].

The differential diagnosis between PD and atypical parkinsonian syndromes is difficult due to the lack of reliable, easily accessible biomarkers. Contents in serum EVs have been shown to be capable of predicting and distinguishing PD from atypical parkinsonian. Jiang et al. showed that α-syn in combination with clusterin in serum-derived NDEVs predictes and differentiates PD from atypical parkinsonism with a promising diagnostic value (AUC = 0.98) [119]. Similarly, Dutta et al. analyzed α-syn levels in serum- or plasma-derived EVs of PD patients, MSA patients, and healthy individuals. They found that α-syn levels are significantly lower in the control group and significantly higher in the MSA group compared with that in the PD group. The ratio of α-syn level in putative ODEVs to that in putative NDEVs is a particularly sensitive biomarker for distinguishing between PD and MSA (AUC = 0.902, sensitivity = 89.8%, specificity = 86.0%). Their data demonstrated that a minimally invasive blood test measuring α-syn level in circulating EVs that can be immunoprecipitated using CNS markers can distinguish between PD patients and MSA patients with high sensitivity and specificity [136].

### EVs as novel biomarkers for the diagnosis of ALS

To date, no definite ALS biomarkers are available. To discover efficient and accessible biomarkers for ALS, studies have been carried out to examine differentially expressed proteins in EVs between ALS and control groups utilizing blood samples from ALS patients. Among them, the level of coronin-1a (CORO1A) is 5.3-fold higher in EVs isolated from plasma of ALS patients than that in the controls [186]. CORO1A level increases with disease progression at a certain proportion in plasma of ALS patients and in the spinal cord of ALS mice. As CORO1A significantly affects ALS pathogenesis, it may be a potential biomarker for ALS [186]. Moreover, in a longitudinal study, plasma-derived EV samples collected from 18 ALS patients aged between 20 and 65 years were analyzed at baseline, and at 1, 3, 6 and 12 months of follow-up [187]. The ratio of neurofilament light chain (NFL) and phosphorylated neurofilament heavy chain (pNFH) was measured by ELISA, and that of TDP-43 was determined by flow cytometry. The ratio of TDP-43 in plasma-derived EVs significantly increased at 3-month and 6-month follow-up. When subclassifying patients into rapid- and slow-progression groups, EV NFL but not pNFH was significantly higher in the rapid-progression group at baseline and at 3-month follow-up [187], indicating NFL in plasma-derived EVs as a biomarker for disease progression. However, further studies are needed to demonstrate the diagnostic power of the aforementioned proteins.

ALS-associated miRNA profiles in EVs from CSF or peripheral blood of patients have also been tested. miR-146a-5p, a miRNA involved in the regulation of synaptic plasticity and inflammatory response through inhibition of synaptotagmin1 and neuroligin1 [188], shows decreased expression in EVs from CSF of ALS patients [189]. However, its diagnostic power remains unknown. Saucier et al. sequenced miRNAs in EVs from plasma of ALS patients, and found differential expression of 22 miRNAs between ALS and controls [190]. Among these miRNAs, miR-15a-5p (AUC = 0.976, sensitivity = 92.9%, specificity = 91.7%) and miR-193a-5p (AUC = 0.844, sensitivity = 80.0%, specificity = 88.9%) show promising diagnostic value for ALS [190]. Similarly, miRNA analysis of L1CAM^+^ NDEVs from ALS patient plasma showed deregulation of 30 miRNAs compared with healthy controls [141]. The deregulated miRNAs are involved in synaptic vesicle-related pathways, four of which are also deregulated in motor cortex tissues of ALS patients [141]. Another study using the same approach identified a potential miRNA fingerprint in L1CAM^+^ NDEVs from plasma of ALS patients (containing miR-146a-5p, miR-199a-3p, miR-151a-3p, miR-151a-5p, and miR-199a-5p) that showed up-regulation in ALS patients compared with healthy controls, while 3 miRNAs (miR-4454, miR-10b-5p, and miR-29b-3p) were down-regulated in ALS [191]. However, the authors did not further validate L1CAM^+^ NDEVs or determine the sensitivity and specificity of these miRNAs regarding the diagnosis of HD.

### EVs as novel biomarkers for the diagnosis of HD

Misfolded proteins or protein aggregates are pathological hallmarks of HD as well, thus studying misfolded proteins or their regulators might be a crucial part in developing biomarkers for HD [192]. Numerous studies have shown that EVs contain mHTT, its fragments, and many other molecules to reflect disease state, which may be potential biomarkers of HD. However, till date, only few studies have analyzed EVs or their contents in seeking for HD biomarkers.

Ananbeh and colleagues reported elevated total HTT levels in plasma-derived EVs of HD patients compared with control donors, as well as in HD pig models compared with control pigs, representing an important initial step towards characterization of EV contents in seeking for HD biomarkers [156]. Afterwards, EVs derived from platelets, a cell type that contains the highest level of mHTT among blood cells [193], were investigated as probable HD biomarker carriers [194]. However, no differences were found in the number of platelet-released EVs between HD patients and healthy controls, and no correlations were found for the number of platelet-released EVs with the age, CAG repeat number, or disease stage of patients [194]. More importantly, mHTT protein is undetectable in EVs released from platelets [194], indicating that platelet-derived EVs might not be able to serve as HD biomarkers. On the other hand, while EV nonprotein contents, such as miRNAs, have been frequently studied for their potential as biomarkers for AD, PD, and ALS, little is known in HD due to the scarcity of relevant studies [192]. Hence, no significant advance has been made in utilizing EVs as potential ALS biomarkers.

Together, the findings discussed above represent important contributions to the identification of EV biomarker candidates for AD, PD, ALS, and HD. More importantly, although isolation of brain cell-derived EVs can be costly, time-consuming, and labor-intensive, a large number of studies have demonstrated that contents (e.g., miRNAs) of brain cell-derived EVs isolated from blood are much more sensitive and specific, compared with blood molecules [2, 195, 196]. However, there are challenges that restrict the application of EVs and their cargos on the diagnosis of NDs. First, advanced technologies are required to minimize contaminants, especially in the plasma, and to clearly validate the key biological/pathological components in EVs. Second, it remains challenging to isolate circulating EVs derived from the brain and identify specific types of brain cells [85, 86], which can be overcome by discovery of more specific markers and development of more innovative separation methodologies. Third, it is important to distinguish between different subtypes of EVs, since reduction of the heterogeneity of EV samples will greatly strengthen diagnostic interpretations. Fourth, as alterations of contents of circulating EVs reflect systemic host responses, studies in large patient cohorts are necessary to clarify the power, sensitivity, and specificity of certain EV contents in the diagnosis of NDs. Therefore, further studies are needed to overcome current challenges and provide a clearer and more comprehensive picture of the utilization of EVs or their contents as standard, routine diagnostic tools for NDs in the clinic.

## EV-based therapeutic strategies in the treatment of NDs

Cells are able to manipulate the molecular composition and function of extracellular matrix *via* secreting EVs to extracellular matrix [197]. EVs transmit signaling molecules through local or distal pathways [198]. Given that EVs can contain and transport toxic molecules and the relatively long-lasting stability of EV contents, EV-based therapeutic strategies are proposed in NDs treatment. As EV-mediated responses can be either disease-promoting or -restraining depending on its contents and states, EVs have been proposed as potential therapeutic targets or agents for ND treatment. Engineered EVs can deliver diverse therapeutic cargos, including short interfering RNAs, antisense oligonucleotides, chemotherapeutic agents, and immune modulators [199]. Importantly, because EVs are components of the native cellular transport system, they would not induce activation of immunogenic responses as external bioactive medications may probably do. Given these properties, engineered EVs are proposed as a potential drug delivery platform for ND therapeutics. Here we summarize recent studies that demonstrate the usage of EVs as therapeutic targets, agents, or drug delivery platforms for ND treatment in cellular and animal studies.

### Pathogenic EVs as targets for the treatment of NDs

Due to the identification of EVs as carriers of pathological molecules during disease progression, pharmacological modification on the release of EVs that contain pathogenic cargos of ND-associated proteins is a common approach for EV-based therapy development. For instance, EVs derived from neurons and activated glial cells were found to carry Aβ, tau, and pathogenically altered miRNAs in AD [10, 83, 84, 87, 111–113]. One study using a transgenic AD mouse model (5×FAD mouse) showed that reducing exosome release by GW4869 decreased total Aβ_1−42_ and the number of plaques in mouse brains, suggesting that reducing exosome release might have therapeutic benefit for AD treatment [95]. Similar results were obtained in an AD in vitro model that blockage of exosome release *via* siRNA for sphingomyelin synthase 2 enhanced Aβ uptake by microglia and significantly suppressed Aβ deposition [89]. However, indiscriminately modifying EV release in the brain may exert undesirable side effects, thus fine-tuning on EVs derived from specific cell types or EVs altered in distinct signaling pathways is in urgent need.

EVs have also been found to carry PD pathogenic cargos such as α-syn and altered miRNAs that mediate disease progression [116, 117, 130, 131]. Studies targeting EVs in PD have revealed promising directions. Indirect modulation of EV release through restoration of the autophagy flux by inhibiting Drp1, the key regulator of mitochondria fission and fusion, attenuates α-syn propagation and aggregation [200]. This study demonstrates that limiting EV release by modulating Drp1 has therapeutic potentials to mitigate α-syn transmission and aggregation in PD, with efficacy shown in both NDEVs and MDEVs [201].

Although many studies have shown promising potential of blocking EV release in animal models of NDs, this strategy remains far from clinical practice. Due to the lack of knowledge on EV biogenesis, it is impossible to manipulate EV secretion without interrupting other biological processes in the cells. The generally accepted approach to blocking EV release is to inhibit the activity of nSMase2 by GW4869, PDDC, and other chemicals [202, 203]. In addition to controlling exosome secretion, nSMase2 and its product ceramide are widely associated with other biological processes, including synaptic vesicle recycling [204], cell death regulation [205], and cell metabolic homeostasis maintenance [206]. Hence, inhibition of nSMase2 activity may inevitably cause many adverse effects. Moreover, GW4869 has been found to reduce exosome release while enhancing MV generation [98]. Without fully dissecting the heterogeneity of EVs under pathological conditions, it would be impossible and meaningless to target key subtypes of EVs with pathogenic potential for treatment of NDs.

### Stem cell-derived EVs as potential therapeutic agents for the treatment of NDs

**Fig. 3:**
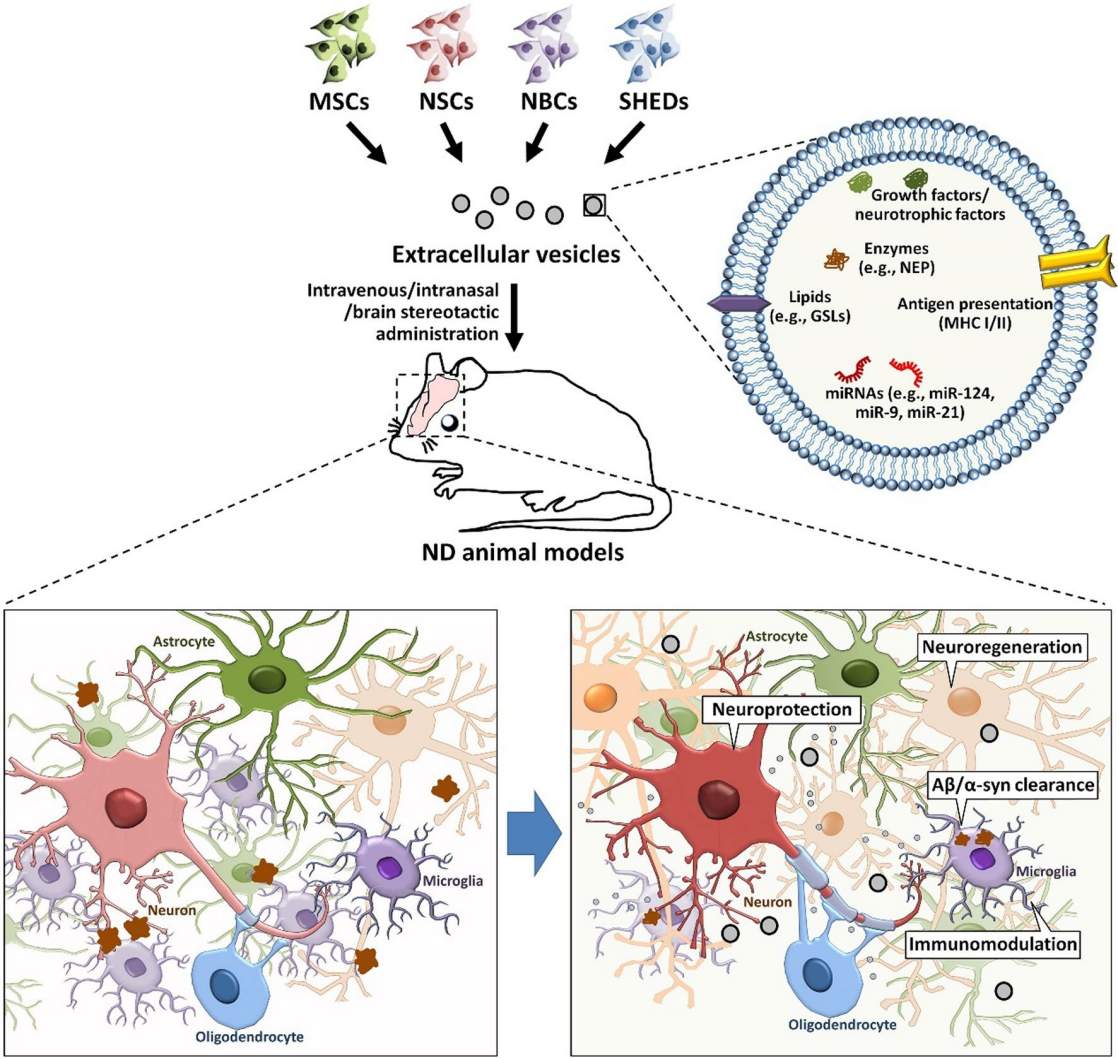
**The therapeutic effects of stem cell-derived EVs on NDs.** To date, the therapeutic effects of EVs derived from MSCs, NSCs, NBCs, and SHEDs have been reported in various animal models of NDs. These stem cell-derived EVs carry Aβ degradation-related enzyme (e.g., NEP) and lipids (e.g., GSLs), growth factors, neurotrophic factors, therapeutic miRNAs. The administration of stem cell-derived EVs therefore improves cognitive/motor function, facilitates Aβ/α-syn clearance, enhances neuroprotection, suppresses neuroinflammation, promotes neuroregeneration of ND animal models

Utilizing EVs as potential therapeutic agents for disease treatment is another area of interest in the field. Recent studies have revealed that, after transplantation, stem cells exert their therapeutic effects by secreting EVs and other factors into the microenvironment *via* a paracrine mechanism. Due to the fact that crossing the BBB is a critical challenge for stem cell therapy, stem cell-derived EV-based therapeutic strategy might be particularly useful for the treatment of NDs (Table 3; Fig. 3).

**Table 3:** Summary of stem cell-derived EVs as potential therapeutic agents for NDs

Cell origin	Disease	Therapeutic cargos	Targets	Administration mode	Models	Outcomes	References
MSC	AD	miR-29	HDAC4	Weekly intravenous injection for 4 weeks	hAPP-J20 mice	Improve cognitive function, decrease Aβ levels, inhibit astrocyte activation.	[208]
MSC	AD	NEP	Aβ	Co-culture	N2a cells	Promote Aβ degradation	[209]
MSC	AD	miR-223	PTEN	Co-culture	Aβ-treated SH-SY5Y cells	Inhibit neuronal cell apoptosis, enhance cell migration	[213]
MSC	AD	–	–	Co-culture	TG2576 mouse-derived neurons	Reduces Aβ levels, attenuates apoptosis, increases neurite outgrowth	[214]
MSC	AD	–	–	Intracerebroventricle injection once per 2 days for 2 weeks	APP/PS1 mice	Mitigate neuroinflammation and neural impairment	[221]
MSC	PD	–	–	SNpc and striatum injection	6-OHDA-injected rats	Attenuate fine motor deficits, protects against TH damage	[216, 218]
MSC	PD	–	–	Diluted in inactivated OP50	Two *C. elegans* models	Reduce α-syn levels	[211]
MSC	PD	–	–	Intravenous injection every 3 days for 8 weeks	6-OHDA-injected rats	Improve the behavioral deficits, reduce dopaminergic neuron loss	[215]
MSC	PD	–	SMAD3, p38 MAPK	Subcutaneous injection into the right forelimb	MPTP-injected mice	Promote angiogenesis	[217]
MSC	PD	–	–	Co-culture	6-OHDA-treated SH-SY5Y cells	Protect neuronal cells from oxidative damage	[223]
MSC	ALS	–	–	Co-culture	Mutant SOD1-NSC-34 cells	Promote cell survival, inhibit apoptotic genes	[219]
MSC	ALS	–	–	Intravenous and intranasal injection	SOD1(G93A) mice	Improve motor performance, inhibit neurodegeneration	[220]
MSC	ALS	–	–	Co-culture	Mutant SOD1-overexpressing NSC-34 cells	Protect NSC-34 cells from oxidative damage and apoptosis	[224]
NSC	AD	–	–	Single retro-orbital vein injection	5×FAD mice	Reduce Aβ deposition, rescue the cognitive defects	[228]
NSC	AD	–	–	Bilateral injection into the lateral ventricles	APP/PS1 mice	Rescue the cognitive defects	[229]
NSC	PD	–	–	Injection into the SN region	6-OHDA-induced mice	Reduce ROS levels and 6-OHDA-induced dopaminergic neuronal loss	[230]
NBC	AD	GSLs	Aβ	Brain infusion for 2 weeks	APP_SweInd_ mice	Decrease Aβ pathologies	[239]
SHED	PD	–	–	Co-culture	6-OHDA-treated neuron	Suppress 6-OHDA-induced apoptosis	[238]

#### Mesenchymal stem cell-derived EVs as potential therapeutic agents for the treatment of NDs

MSCs are the most commonly investigated stem cells for therapies due to their ability of damage repair and inflammation modulation. Mounting in vitro and in vivo studies have demonstrated promising effects of MSCs on neurological recovery, immunomodulation, and neo-angiogenesis in various NDs [207]. In recent years, MSC-derived EVs have attracted much attention since they exhibit similar therapeutic effects as their parental cells in treating NDs and have multiple advantages including negligible immunogenicity, more flexible administration strategies, and convenient content and surface modifications [166]. Emerging evidence has suggested that the MSC-derived EVs achieve their therapeutic effects *via* multiple mechanisms.

MSC-derived EVs facilitate the degradation of pathogenic proteins, and have been shown to attenuate Aβ expression while increasing expression of genes related with memory and neural synaptic function in both cell and animal models of AD [208]. These alterations, in turn, elevate brain glucose metabolism and reverse cognitive dysfunctions in AD transgenic mice [208]. Katsuda and colleagues reported the existence of neprilysin (NEP), one of the most pivotal Aβ-degrading enzymes, in adipose MSC-derived EVs [209]. In cultured cells, NEP-loaded EVs reduce levels of both released and intracellular Aβ in neuroblastoma cells (NBCs), demonstrating the beneficial significance of adipose MSC-derived EVs for AD [210]. Likewise, bone marrow MSC-derived EVs largely reverse dopaminergic neurodegeneration in a *C. elegans* model of PD through decreasing α-syn aggregates, suggesting that the MSC-derived EVs facilitate degradation of pathogenic proteins in PD [211].

MSC-derived EVs also exert anti-apoptosis and pro-survival effects [212–216]. Wei et al. demonstrated that miR-223-enriched MSC-derived EVs inhibit neuronal cell apoptosis and enhance cell migration in an AD cell model *via* PTEN and PI3K-Akt pathways [213]. Lee et al. also demonstrated that EVs secreted by adipose MSCs reduce β-amyloidosis and neuronal apoptosis in AD transgenic mice and enhance axonal growth in the brains of AD patients [214]. Decreased expression of p53, Bax, pro-caspase-3 and cleaved-caspase-3, and increased expression of Bcl-2 have been found following treatment with adipose MSC-derived EVs in AD transgenic mice [214]. This study reflects the pro-survival effects of MSC-derived EVs against Aβ-triggered neuronal dysfunction and neural loss [214]. In addition, recent studies have shown that pretreatment with MSC-derived EVs dampened 6-OHDA-stimulated SH-SY5Y cell apoptosis through boosting autophagy for neural protection [215, 216]. In 6-OHDA-injected rats, transplanted EVs cross the BBB, diminish apoptosis of dopaminergic neurons, and meanwhile enhance dopamine levels in the striatum [215]. In MPTP-treated mice, Xue et al. found that MSC-derived EVs stimulate angiogenesis of human brain microvascular endothelial cells (HBMECs) following enhancing the expression of intercellular adhesion molecule-1 (ICAM-1) and restoring the 1-methyl-4-phenylpyridinium (MPP^+^)-induced damage to endothelial cells [217]. Indeed, MSC-derived EVs trigger HBMEC angiogenesis through up-regulation of ICAM1 by provoking the SMAD3 and P38MAPK signaling pathways [217]. Moreover, intraperitoneal injection of EVs notably increases TH^+^ dopaminergic neurons in substantia nigra (SN) and up-regulates CD31 expression in the corpus striatum of treated mice, leading to recovery of these animals [217]. Reports also showed that the desired pro-survival effects of MSC-derived EVs are, to a large extent, mediated by various biologic molecules in MSC-derived secretomes, including SDF-1, growth factors (BDNF, VEGF and GDNF), MMP2, heat shock protein 27, and semaphorin 7a in 6-OHDA-injected rats [218]. Moreover, scientists demonstrated that adipose MSC-derived EVs play a neuroprotective role in ALS models in vitro [219]. They discovered 189 proteins in adipose MSC-derived EVs that contribute to cell adhesion and negative modification of the apoptotic pathways. Further analysis revealed that the EV therapy suppresses the expression of pro-apoptotic proteins Bax and cleaved caspase-3 and conversely increases the expression of anti-apoptotic protein Bcl-2 in ALS in vitro models [219]. Intravenous and intranasal administration of adipose MSC-derived EVs in the SOD1G93A mouse model of ALS protects lumbar spinal cord motor neurons from neurodegeneration presumably through suppressing glial cell functions up to 17 weeks post-transplantation [220].

MSC-derived EVs exert neuroprotective effects through reversing brain inflammation. Wang et al. found improved cognitive behaviors / synaptic transmission and suppression of expression of pro-inflammatory iNOS after MSC-derived EV administration in AD mice, and that down-regulation of iNOS expression indeed rescues neural function impairment in vivo [221]. In addition, MSC-derived EVs up-regulate the expression of anti-inflammatory factors such as IL-10 and tissue inhibitor matrix metalloproteinase 1 in activated microglia, implying an important role of MSC-derived EVs in initiating anti-inflammatory responses in AD mice [222].

Moreover, MSC-derived EVs show promising antioxidant effects in various ND models. For example, Chierchia et al. found that MSC-derived EVs elicit antioxidant effects by elevating Sirt3 expression in 6-OHDA-treated SH-SY5Y cells, which led to further neuroprotective effects in vivo [223]. Furthermore, a recent in vitro study revealed that adipose MSC-derived EVs protect NSC-34 cells that overexpress human SOD1(G93A) from oxidative stress and rescue NSC-34 cells from apoptosis, suggesting that MSC-derived EVs function as potential antioxidants in treating ALS [224].

Taken together, MSC-derived EVs have been shown to alleviate disease phenotypes in various cell and animal models through various mechanisms. However, the therapeutic effects of MSC-derived EVs have to be confirmed in clinical studies, which is currently under investigation in China and many other countries (ClinicalTrials.gov Identifier: NCT04388982).

#### Neural stem cell (NSC)-derived EVs as potential therapeutic agents for the treatment of NDs

Unlike MSCs, NSCs are a population of endogenous stem cells in the CNS that play a crucial role in the neural development and repair as they differentiate into both neurons and neurogliocytes [225]. To date, the therapeutic roles of NSC-derived EVs have been studied in various models of NDs and acute neural injury, and encouraging results have been obtained [17, 226]. Importantly, studies have demonstrated that NSC-derived EVs have better effects in improving neural function recovery than MSC-derived EVs [227]. These findings suggest that the NSC-derived EVs inherit the great neurogenic/neuroregenerative potential from their parent cells, making them potential therapeutics for NDs.

Till now, investigations on the therapeutic effects of NSC-derived EVs on NDs mainly focus on AD. Multiple independent groups have reported that NSC-derived EVs rescue cognitive defects in different AD animal models including 5×FAD and APP/PS1 transgenic mice [228, 229]. Different pathological and molecular mechanisms of neurofunction restoration by NSC-derived EVs in NDs have been unveiled. Similar to MSC-derived EVs, NSC-derived EVs may also reduce the burdens of key pathological molecules in NDs. A single injection of human NSC-derived EVs in the retro-orbital vein significantly reduces Aβ deposition in the brains of 5×FAD transgenic mice [228]. However, conflict results have been reported that the lateral ventricle injection of NSC-derived EVs does not alter Aβ burden in APP/PS1 transgenic mice [229]. Thus, the effects of therapies based on NSC-derived EVs on Aβ deposition remain an open question. NSC-derived EVs also achieve their therapeutic effects through immunomodulation and neuroprotection. After intravenous injection, NSC-derived EVs inhibit activation of microglia and excessive expression of pro-inflammatory cytokines in AD mouse brains highly likely through the delivery of miR-124 and other inflammation-regulatory miRNAs [228]. Meanwhile, NSC-derived EVs also restore the levels of memory-related synaptic proteins and improve synaptic morphology in the cortex of AD mice by promoting mitochondrial function and decreasing oxidative damage, suggesting promising neuroprotective effects of NSC-derived EVs [229]. Similar results have been obtained in 6-OHDA-induced PD model that administration of NSC-derived EVs down-regulates pro-inflammatory signals and decreases the 6-OHDA-induced dopaminergic neuronal loss in vivo [230]. NSC-derived EVs also enhance neuroregeneration in 5×FAD mouse brains including increasing the NSC pool and facilitating NSC differentiation into neuronal lineage, presumably through transferring miRNAs (e.g., miR-9, and miR-21) and proteins (e.g., growth factors) to endogenous NSCs [17]. Moreover, NSC-derived EVs reverse AD-caused BBB disruption in vitro and in vivo [231]. Together with the finding that NSC-derived EVs promote angiogenesis in CNS injury [232], cerebrovascular regulation could be an important therapeutic effect of NSC-derived EVs.

Notably, although NSC-derived EVs exhibit outstanding therapeutic effects, NSCs also have disadvantages including ethical/religious concerns and problematic logistics of acquiring fetal tissues, restricting mass production of EVs [233]. Our recent studies have generated NSC-like cells using cell reprogramming approach [234]. In this approach, somatic cells like fibroblasts and astrocytes are directly reprogrammed into induced NSCs (iNSCs) that exhibit comparable proliferation/renewal and multipotent differentiation capacities [235]. The iNSC-derived EVs exhibit comparable or even better performance in enhancing the proliferation, migration, and differentiation of NSCs *in vitro via* transferring growth factors including EGF, FGF2, and IGF [236]. The iNSC-derived EVs also significantly inhibit apoptosis of NSCs induced by oxidative stress or starvation [237]. More importantly, intravenous administration of iNSC-derived EVs promotes recovery of neurofunction and neural tissue regeneration, and suppresses neuroinflammation and neuronal injury in a stroke model [17] and an AD mouse model (unpublished data), presumably through activation of the MEK/ERK signaling pathway. Therefore, both NSC- and iNSC-derived EVs display great therapeutic effects in cell and animal models of NDs, implicating the equal necessity to evaluate the potential of NSC- and iNSC-derived EVs for clinical application, compared to MSC-derived EVs.

#### Potential therapeutic effects of EVs derived from other stem cell types on NDs

Besides the aforementioned two types of stem cells, there are other types of stem cells that have been utilized as EV producer for ND treatment [238]. For instance, NBC-derived EVs have been reported to trap Aβ and facilitate Aβ internalization into brain-resident phagocyte microglia [239]. This finding demonstrates that intracerebrally administered EVs utilize membrane glycosphingolipids (GSLs) to act as Aβ scavengers and suggests a role for NBC-derived EVs in Aβ clearance in the brain [239]. Furthermore, scientists have found that EVs derived from the microcarrier-cultured stem cells from the dental pulp of human exfoliated deciduous teeth (SHEDs) suppress the 6-OHDA-induced apoptosis of dopaminergic neurons [238], probably through reducing the sensitivity of dopaminergic neurons to the 6-OHDA-induced oxidative stress [240]. Notably, EVs derived from SHEDs under standard culture conditions do not exert similar anti-apoptotic effect, indicating that culture conditions have a crucial influence on EV function.

Hence, although multiple types of stem cells have been utilized to generate EVs as potential therapeutics of NDs, much more investigations are urgently required to investigate the therapeutic effects of EVs derived from more types of stem cells, to fully unravel the underlying mechanisms of alleviation of ND phenotypes by stem cell-derived EVs, and to develop a standard for preparing pharmaceutical-grade EVs in order to accelerate clinical applications of these EVs in ND treatment.

### Engineered EVs as a potential drug delivery platform for treatment of NDs

**Fig. 4:**
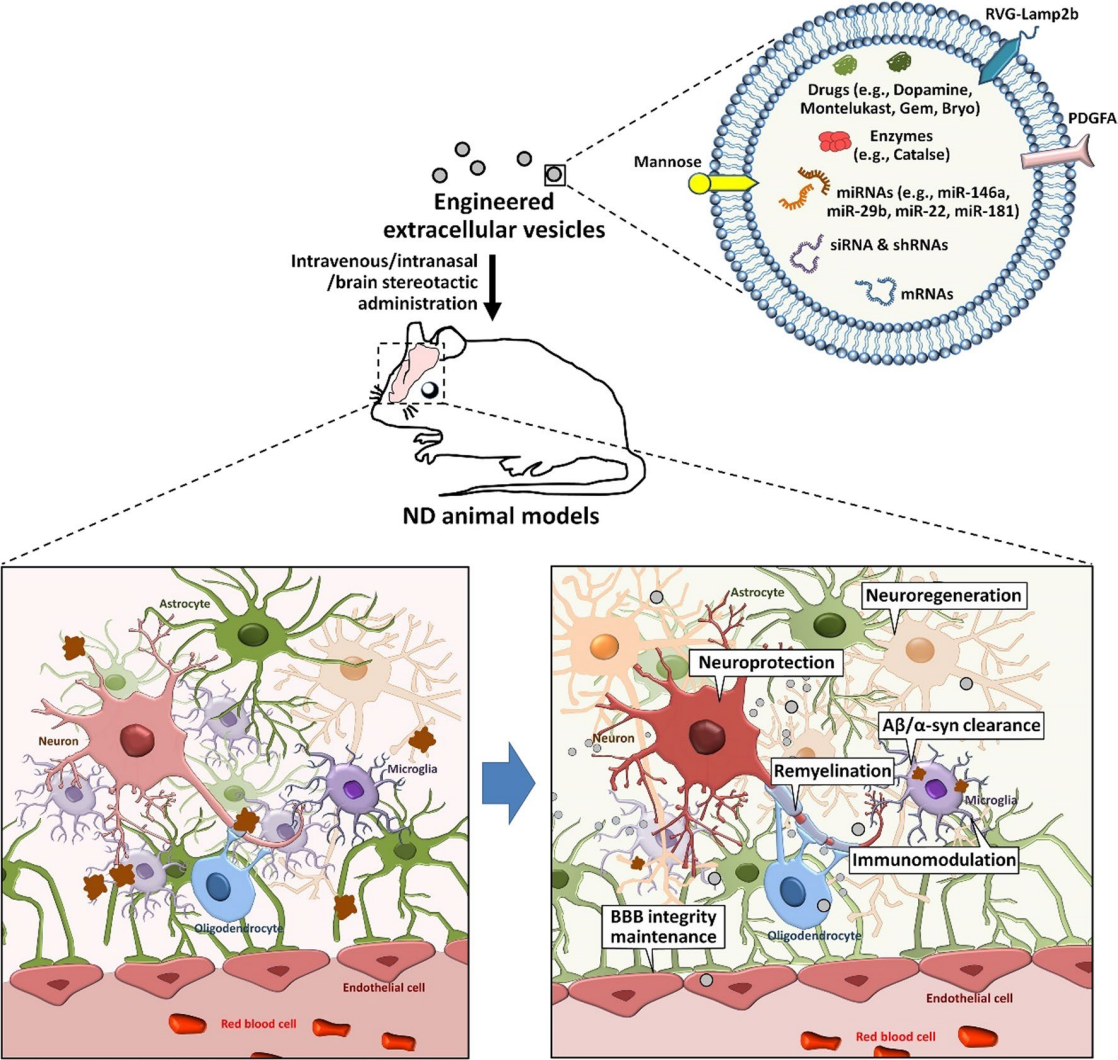
**The therapeutic effects of engineered EVs on NDs.** EVs have been utilized as a drug delivery platform for the treatment of NDs. Therapeutic cargos including shRNA/siRNA/miRNAs that target ND-related genes, mRNAs that express Aβ degradation enzymes, and therapeutic drugs can be specifically loaded into EVs *via* transfection or physical strategies. Moreover, through being decorated with RVG, mannose, or PDGFA on the surface, EVs are further conferred targeting capacity to the CNS, microglia, and OPCs, respectively. These engineered EVs reach their target cells to facilitate Aβ clearance, mitigate oxidative stress, protect neuronal cells, inhibit neuroglial activation, promote remyelination, and restore BBB integrity, thus alleviating behavioral phenotypes of ND animal models

Mounting studies have demonstrated that engineered EVs can be an effective platform for delivery of drugs [241, 242]. As described previously in this review, EVs can permeate membranes including the BBB, indicating them as an effective platform for the delivery of drugs to the CNS [243, 244]. Moreover, engineered EVs may also be able to target specific recipient cells for site-specific delivery [201, 245], suggesting feasibility of intravenous or intranasal delivery approaches that avoid neurosurgery (Table 4; Fig. 4).

**Table 4:** Summary of EVs with drug delivery capacity for the treatment of NDs

Cell/tissue origin	Disease	Surface modification	Loaded cargos	Therapeutic Targets	Administration mode	Models	Outcomes	References
Dendritic cell	PD	RVG	α-syn-siRNA	α-syn	Intravenous injection	α-Syn Tg mice	Reduce intraneuronal α-syn aggregates	[247]
Dendritic cell	PD	RVG	α-syn-shRNA	α-syn	Intravenous injection	Syn PFFs-injected mice	Reduce α-syn aggregation, decrease neuronal death, alleviate PD symptoms	[249]
U87 cell	HD	–	Htt siRNA	Htt	Infusion into the striatum for 7 days	Wild type mice	Reduce Htt expression	[248]
MSC	AD	RVG	miR-146a	NF-κB pathways	Intracerebroventricular injection for 2 times	APP/PS1 mice	Inhibit pro-inflammatory responses of astrocytes and microglia	[167]
MSC & 293T cell	AD	–	miR-29b	BACE1, BIM	Bilateral injection into hippocampal CA1 region	Aβ-treated model rats	Enhance spatial learning and memory	[251]
MSC	AD	–	miR-22	GSDMD	Intravenous injection every 7 days	APP/PS1 mice	Enhance behavioral performance, repress neuroinflammation	[252]
MSC	PD	–	miR-188-3p	CDK5, NLRP3	Intravenous injection for 5 days	MPTP-induced PD mice	Suppress autophagy and pyroptosis	[253]
293T cell	PD	–	Catalase mRNA	ROS	Implantation of EV producers into the brains	6-OHDA-injected mice	Protect neurons against neurotoxicity, inhibit neuroinflammation	[246]
Macrophage	PD	–	Redox catalase	ROS	Intranasal injection for 10 times every other day	6-OHDA-injected mice	Eliminate ROS and display neuroprotective effects	[254]
Macrophage	AD	Mannose	Gemfibrozil	Aβ	Intraperitoneal injection for 7 consecutive days	Aβ-injected mice	Promote Aβ clearance by microglia, improve learning and memory ability	[267]
Blood	PD	–	Dopamine	Dopamine receptors	Intravenous injection	6-OHDA-injected mice	Improve dopaminergic neurons, ameliorate PD phenotype	[255]
NSC	MS	PDGFA	Montelukast	GPR17	Intranasal injection every day for 2 weeks	Cuprizone-treated mice	Promote myelin regeneration	[257]
NSC	MS	–	Bryostatin-1	GPR17	Intravenous injection	Cuprizone-treated mice	Promote myelin regeneration	[256]

#### Engineered EVs with modified cargos for the treatment of NDs

To date, EVs have been successfully used for the delivery of therapeutically active molecules, including RNAs, proteins, and pharmaceutical compounds to the brain [241, 242]. For instance, therapeutic genetic materials that could regulate gene expression have been transported to the brain by EVs to alter ND progression. Scientists have reported that therapeutic catalase mRNAs delivered by engineered EVs alleviate neurotoxicity and neuroinflammation in both cell and animal models of PD, indicating the potential value of EVs for delivery of genetic materials in therapeutic applications [246]. The therapeutic potential of EV-based siRNA delivery for NDs has also been reported. One research group systemically injected modified EVs containing α-syn siRNA into the S129D α-syn transgenic mice and found reduced mRNA and protein levels of α-syn in mouse brain [247]. Another group reported that EVs carrying hydrophobically modified siRNA to the CNS efficiently targeted mHtt mRNA in a HD mouse model [248]. Though therapeutic siRNAs can be efficiently transported by EVs to the CNS [245], their effectiveness is limited in ND treatment due to short time of efficacy and poor bioavailability in systemic circulation. Therefore, scientists investigated the efficacy of EV-delivered shRNAs. They found that α-syn shRNAs delivered by EVs reduce α-syn aggregation, decrease dopaminergic neuronal death, and alleviate PD symptoms in mice [249]. These studies again support the potential of EVs as a drug delivery platform for genetic modulators, such as siRNAs and shRNAs, into the CNS for therapeutic benefits. On the other hand, EV delivery has been demonstrated to increase the stability of various RNA-based therapies for NDs, as they protect RNAs from degradation [250]. Besides siRNAs and shRNAs, miRNAs with therapeutic effects have also been loaded into EVs to treat NDs. In a rat model of AD, bilateral hippocampal injection of miR-29-enriched MSC-derived EVs alleviates the pathological impacts of Aβ peptide and improves spatial learning and memory by suppressing the expression of BACE1, suggesting an inhibitory effect of MSC-derived EVs on Aβ formation [251]. Similarly, miR-146a has been packaged into bone marrow MSC-derived EVs, which down-regulates NF-κB pathways in astrocytes and restores astrocytic activation, ultimately leading to improved synaptogenesis and amelioration of cognitive deficits in AD mice [167]. Adipose MSC-derived EVs specifically loaded with miR-22 enhance the motor and memory capability of AD mice by inhibiting inflammatory factors, down-regulating pyroptosis, and improving neural survival [252]. Further studies demonstrated that MSC-derived EVs loaded with miR-188-3p exhibit anti-inflammatory and anti-apoptotic effects in PD animal models through inhibiting NLRP3-induced inflammation and cyclin-dependent kinase 5-induced autophagy [253].

Besides, EVs have been modified to package natural enzymes for the clearance of pathogenic molecules in NDs. After being transfected with catalase-encoded plasmid DNA, mouse macrophages release EVs preloaded with redox catalase [254]. These EVs significantly increase the viability of 6-OHDA-pretreated PC12 cells as they decrease ROS levels in activated macrophages, suggesting that they reduce neuroinflammation by decreasing ROS in activated microglia [254]. Indeed, in vivo experiments confirmed that catalase-preloaded EVs reduce microglial activation and increase survival of dopaminergic neurons in 6-OHDA-intoxicated mice [254].

Apart from transporting genetic materials or modulators, various in vitro and in vivo studies have suggested EVs as promising vehicles for direct delivery of therapeutic agents for ND treatment. One study reported that EVs isolated from human blood and preloaded with saturated dopamine solution are able to cross the BBB for dopamine delivery into the CNS *via* interactions with transferrin and the transferrin receptor [255]. Another in vivo study showed that dopamine-preloaded EVs display greater therapeutic efficacy and lower toxicity than intravenously delivered free dopamine [201]. Furthermore, NSC-derived EVs have been utilized to package montelukast and bryostatin-1, two drugs for MS [256, 257]. Results showed that the EV-based delivery of drugs to lesion areas in cuprizone-treated mice, an animal model of MS, protects the myelin sheath and promotes remyelination, suggesting EVs as a novel drug delivery platform with great potential for treatment of NDs [256, 257].

#### Engineered EVs with modified surface for targeted therapy of NDs

Although stem cell-derived EVs are proposed as potential treatment tools that have demonstrated beneficial efficacy in a number of NDs [258, 259], further investigations and clinical trials are required to confirm the benefits of therapeutic application of EVs in NDs. Until now, mounting evidence supports that modification of EVs in order for specific targeting may hold substantial therapeutic benefits for NDs [245, 260–264].

Currently, the most commonly used strategy to confer brain-targeting capacity to exosomes is to conjugate the CNS-specific rabies viral glycoprotein (RVG) peptide (YTIWMPENPRPGTPCDIFTNSRGKRASNG) with an exosomal membrane protein Lamp2b [245]. Through transfection of plasmids encoding the RVG-Lamp2b constructs, cells release RVG-expressing exosomes that could be used to deliver desired molecules into the CNS. Multiple studies have utilized this approach to deliver siRNAs, shRNAs, and miRNAs into the brain through dendritic cell- and MSC-derived exosomes, and obtained outstanding outcomes in alleviating AD and PD phenotypes in vivo [167, 247, 249]. It is noteworthy that RVG may not help exosomes to cross the BBB directly and target the CNS through retrograde transport from peripheral nerves [261, 265]. Instead, RVG may confer the BBB penetration capacity to exosomes through direct interaction with nicotinic acetylcholine receptors expressed on endothelial cells [261, 266]. A similar strategy that expresses the cyclo(Arg-Gly-Asp-D-Tyr-Lys) peptide [c(RGDyK)] on exosomal surface, which has high affinity to the integrin αvβ3 in reactive cerebral vascular endothelial cells, successfully facilitates accumulation of modified exosomes in brain lesions compared to the undamaged tissue on the contralateral side of the brain in an in vivo ischemic model [262].

Moreover, scientists have bioengineered EVs to target specific types of cells in the CNS. For instance, to address the inefficient clearance of Aβ caused by abnormal lysosomal function in microglia in AD, EVs are bioengineered to target microglia by adding mannose on EV surface [267]. Mannose-expressing EVs specifically bind to mannose receptor (CD206), a microglial enriched protein, therefore enhancing the uptake of EVs by microglia. Through this approach, EVs deliver gemfibrozil to restore the lysosomal activity of microglia, accelerate lysosome-mediated clearance of Aβ in microglia, and successfully improve the learning and memory ability of AD mice [267]. Similarly, PDGFA can be expressed on engineered EVs that exhibit excellent affinity to oligodendrocyte progenitor cell (OPC) surface receptor PDGFRα [257]. PDGFA-expressing EVs therefore transfer montelukast to OPCs to promote oligodendrocyte generation and myelin regeneration, resulting in mitigation of MS-like phenotypes in a cuprizone-induced demyelination animal model [257].

To summarize, EVs orchestrate various events that facilitate recovery and regeneration in neurodegenerative conditions. Many efforts have been made to improve the homing property of EVs to convey therapeutic agents to brain sites and potentiate recovery. Merging the intrinsic attributes of EVs with a targeted medicine is proposed as a novel therapeutic strategy that may exert a profound influence on the future of ND treatment. However, in view of translation into clinic, some technical challenges still need to be solved. One important challenge is how to enhance BBB penetration and targeting potential of EVs. To date, the mechanisms underlying EV crossing the BBB remain controversy and multiple theoretical routes have been proposed [268]. Based on these theories, EVs can be macropinocytosed or transcytosed into the MVBs of endothelial cells through the endocytic pathway, and then traffick from the MVB to the plasma membrane as neoformed exosomes [268, 269]. EVs may also cross the BBB through the paracellular pathway when BBB integrity is disturbed under pathological conditions [269]. Thus, more studies that sort out the BBB penetration mechanisms will dramatically increase the number of EVs that reach the CNS after intravenous or even oral administration. Moreover, the cargo loading efficiency of EVs remains limited. Intrinsically packed natural molecules (e.g., proteins and nucleic acids) significantly increase the difficulty of desired cargo loading, resulting in a much lower cargo loading efficiency for these EVs than unpacked synthetic liposomes [270, 271]. Besides, other technical issues like quality control due to the high heterogeneity of EVs, hard expedition towards industrial manufacturing, high cost of production and storage also impede the application of EVs for drug delivery [270].

## Conclusions and future perspectives

In summary, numerous studies have demonstrated a tight association of EVs with NDs including but not limited to the direct delivery of pathogenic molecules, the modulation of inflammatory responses of immune cells, the regulation of neuronal cell function and viability, and manipulation of BBB integrity. Inspiringly, the aforementioned findings have suggested great values in translational medicine. With the help of newly developed immunoprecipitation approaches, EVs derived from specific types of brain cells could be purified and certain cargos within these EVs have been reported to be outstanding biomarkers for the diagnosis and prognosis of NDs, providing novel perspectives to realize early diagnosis, a key step for effective prevention of irreversible neurodegeneration. Moreover, drugs targeting pathogenic EVs as well as EVs with therapeutic effects or drug delivery capacity have demonstrated promising therapeutic potential in cellular and animal models of NDs, including mitigating neurofunction impairment, alleviating neuroinflammation and neurotoxicity, and mitigating neurodegeneration and neuronal loss. With extensive investigations, more pathological/beneficial roles of EVs in ND pathogenesis and the underlying mechanisms could be unveiled, dramatically expanding our understanding of EVs within the CNS and shedding light on the development of EV-based therapeutic strategies for more precise diagnosis and more effective treatment of NDs.

## Data Availability

Not applicable.

## Abbreviations

α-syn: α-synuclein
Aβ: Amyloid-beta
AD: Alzheimer’s disease
ADEV: Astrocyte-derived extracellular vesicle
ALS: Amyotrophic Lateral Sclerosis
AUC: Area under the curve
BBB: Blood-brain barrier
CNS: Central nervous system
CORO1A: Coronin-1a
CSF: Cerebral spinal fluid
DPRs: Dipeptide repeat proteins
EBV: Epstein Barr virus
ELISA: Enzyme-linked immunosorbent assay
EV: Extracellular vesicle
GSLs: Glycosphingolipids
GST: Glutathione S-transferase
GWAS: Genome-wide association studies
HBMEC: Human brain microvascular endothelial cell
HD: Huntington’s Disease
HIV: Human immunodeficiency virus
HPV: Human papilloma virus
ICAM-1: Intercellular adhesion molecule-1
IL: Interleukin
ILV: Intraluminal vesicle
iNSC: Induced neural stem cell
LMP1: Latent membrane protein 1
MAC: Membrane attack complex
MCI: Mild cognitive impairment
MDEV: Microglia-derived extracellular vesicle
MPP: 1-methyl-4-phenylpyridinium
MSA: Multiple system atrophy
MSC: Mesenchymal stem cells
MS: Multiple sclerosis
MV: Microvesicle
MVBs: Multivesicular bodies
NBC: Neuroblastoma cell
NCAM: Neural cell adhesion molecule
ND: Neurodegenerative disease
NDEV: Neuron-derived extracellular vesicle
NDUFS3: NADH ubiquinone oxidoreductase core subunit S3
NEP: Neprilysin
NFL: Neurofilament light chain
NSC: Neural stem cell
nSMase2: Neutral sphingomyelinase2
ODE: Oligodendrocyte-derived extracellular vesicle
OPC: Oligodendrocyte progenitor cells
p-Tau: Phosphorylated Tau protein
PD: Parkinson’s disease
PMEL: Premelanosome protein
pNFH: phosphorylated neurofilament heavy chain
PrP: Prion protein
RISC: RNA-induced silencing complex
ROC: Receiver operating characteristic
ROS: Reactive oxygen species
RVG: Rabies viral glycoprotein
scFvs: Single-chain fragment variable antibodies
SDF-1: Stromal cell-derived factor-1
SDHB: Succinate dehydrogenase complex subunit B
SEMA3A: Semaphorin 3 A
SN: Substantia nigra
SOD: Superoxide dismutase
T2DM: Type 2 diabetes mellitus
TDP-43: TAR DNA-binding protein-43
TNF-α: Tumor necrosis factor-alpha
UTR: Untranslated region
